# Single‐cell atlas of peripheral blood by CyTOF revealed peripheral blood immune cells metabolic alterations and neutrophil changes in intracranial aneurysm rupture

**DOI:** 10.1002/mco2.637

**Published:** 2024-07-15

**Authors:** Xiaolong Ya, Chenglong Liu, Long Ma, Peicong Ge, Xiaoxue Xu, Zhiyao Zheng, Siqi Mou, Rong Wang, Qian Zhang, Xun Ye, Dong Zhang, Yan Zhang, Wenjing Wang, Hao Li, Jizong Zhao

**Affiliations:** ^1^ Department of Neurosurgery, Beijing Tiantan Hospital Capital Medical University Beijing China; ^2^ China National Clinical Research Center for Neurological Diseases Beijing China; ^3^ Department of Core Facility Center Capital Medical University Beijing China; ^4^ Department of Neurosurgery, Peking Union Medical College Hospital Chinese Academy of Medical Sciences and Peking Union Medical College Beijing China; ^5^ Medical School University of Chinese Academy of Sciences Beijing China; ^6^ Department of Neurosurgery Beijing Hospital Beijing China; ^7^ Beijing Institute of Hepatology, Beijing YouAn Hospital Capital Medical University Beijing China

**Keywords:** CyTOF, heterogeneity of neutrophils, immune metabolism, intracranial aneurysms

## Abstract

Previous studies have found that the peripheral immune environment is closely related to the occurrence and development of intracranial aneurysms. However, it remains unclear how the metabolism of peripheral blood mononuclear cells (PBMCs) and the composition of polymorphonuclear leukocytes (PMNs) changes in the process of intracranial aneurysm rupture. This study utilized cytometry by time of flight technology to conduct single‐cell profiling analysis of PBMCs and PMNs from 72 patients with IAs. By comparing the expression differences of key metabolic enzymes in PBMCs between patients with ruptured intracranial aneurysms (RIAs) and unruptured intracranial aneurysms, we found that most PBMCs subsets from RIA group showed upregulation of rate‐limiting enzymes related to the glycolytic pathway. By comparing the composition of PMNs, it was found that the proinflammatory CD101+HLA DR+ subsets were increased in the RIA group, accompanied by a decrease in the anti‐inflammatory polymorphonuclear myeloid‐derived suppressor cells. In conclusion, this study showed the changes in the peripheral immune profile of RIAs, which is helpful for our understanding of the mechanisms underlying peripheral changes and provides a direction for future related research.

## Graphical abstract

1. *Immune cell metabolism*: Explore the metabolic changes of peripheral immune cells after intracranial aneurysm rupture.
2. *Neutrophil heterogeneity*: To explore the changes in the composition and function of peripheral blood neutrophils after intracranial aneurysm rupture.
3. *Therapeutic insights*: Understanding the mechanism of peripheral immune dysfunction in patients after rupture.

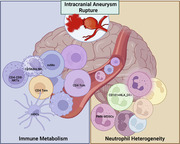

## 1 INTRODUCTION

Intracranial aneurysm (IA) is the main cause of nontraumatic subarachnoid hemorrhage (SAH).^1^, ^2^ The development and rupture of IAs are related to various factors, among which inflammation is considered a key driving factor.^3^ In addition to various immune components directly participating in the progression of local lesions, many research results indicated that changes in the peripheral immune environment were also important factors influencing the rupture and poor prognosis of aneurysms.^4^, ^5^ However, due to the complexity and heterogeneity of immune cells, there is still a lack of detailed understanding of the peripheral immune composition and changes in ruptured intracranial aneurysm (RIA).

Studies have shown that the peripheral immune environment plays an important mediating role in the development of IAs caused by some chronic inflammatory diseases.^6^, ^7^ Its fluctuations may exacerbate the disruption of the local immune environment, leading to the rupture of intracranial aneurysms. Peripheral blood mononuclear cells (PBMCs), as the main builders of the peripheral immune environment, have functional changes that can significantly affect the peripheral immune environment.^8^ Previous high‐dimensional analyses have found abnormal activation and functional dysfunction of various PBMC subsets in the peripheral blood of patients with IAs.^9^, ^10^ Studies suggested that the behavior and function of immune cells in immune responses were mainly regulated by their own metabolic patterns.^11^, ^12^ Abnormal activation and functional changes are closely related to specific metabolic shifts. Aberrant immune cell metabolic reprogramming can disrupt immune homeostasis, leading to various diseases.^13^ Recently, the successful development of small molecule metabolism‐targeted drugs has made it possible to regulate abnormal immune cell metabolic patterns.^14^ Restoring immune homeostasis by interfering with cellular metabolic processes is considered as a new therapeutic strategy. Therefore, conducting related research is conducive to understanding the metabolic pattern shifts behind the functional changes of peripheral immune cells in the RIAs, providing intervention targets for precision metabolic therapy.

Clinical studies have found that the elevation of neutrophils in peripheral blood is closely related to the instability of IAs.^5^ Furthermore, upregulation of neutrophil elastase expression has been observed in the peripheral blood of IA patients with high risk of rupture.^15^, ^16^ These results all suggested that neutrophils in peripheral blood played an important role in the rupture of IAs. Transcriptome analysis of peripheral blood polymorphonuclear cells (PMNs) revealed heterogeneous transcriptional characteristics of neutrophils, with some features closely related to the development of IAs.^17^ Recent studies have also revealed significant heterogeneity in neutrophils, which were traditionally considered a homogeneous population.^18^, ^19^ Neutrophil subsets with different characteristics play different roles in the development of the same disease.^20^ The concept of heterogeneity suggests that peripheral neutrophils may play a more complex role in the progression of IA lesions. Therefore, detailed analysis of peripheral blood neutrophils at the single‐cell level is beneficial for understand their role in the mechanism of aneurysm rupture. It helps identify the key cell subsets leading to the lesion and provides direction for further development of precision immune therapy.

Cytometry by time of flight (CyTOF) offers the advantage of high‐dimensional single‐cell analysis, allowing for comprehensive characterization of cell populations based on multiple parameters simultaneously.^21^ Previous studies have utilized these advantages to develop the single‐cell metabolism regulatory component analysis technique.^11^, ^22^, ^23^ This method employs high‐dimensional antibodies to quantify proteins regulating metabolic pathways. It enables direct comparative analysis of the metabolic states of all immune cell subtypes without the need for preselection.^11^ Moreover, specific surface and functional markers reacting with distinct subgroups have been used to identify different PBMCs and PMNs cell subgroups and their functional states.^24^ In this study, we used CyTOF technology to comprehensively profile the single‐cell immune landscape of peripheral blood from IAs patients. By comparing the differences between patients with unruptured intracranial aneurysms (UIAs) and RIAs, we further explore the changes in peripheral immune of RIAs from the perspectives of PBMC metabolic alterations and neutrophil heterogeneity.

## 2 RESULTS

To comprehensively depict the peripheral immune landscape of patients with IAs, we conducted analyses from two aspects: PBMCs and PMNs. In analyzing PBMCs, we first employed gating strategies to isolate common immune cell types (T, B, NK, monocytes) from PBMCs, and then performed separate analyses on each cell type (detailed procedures in Figure S1). For each cell type analysis, we first annotated various subsets based on the distribution of surface markers, and then the differences of immune cells between the two groups were compared in terms of the proportion, the functional and metabolic molecules.

### 2.1 PD1− CD4 Tem and T‐bet+ CD4 Tcm from RIA showed upregulated expression of the glycolysis‐related rate‐limiting enzymes PKM2 and LDH, whereas CD56^dim^ DN‐NKT exhibited upregulation of multiple metabolic markers

**FIGURE 1:**
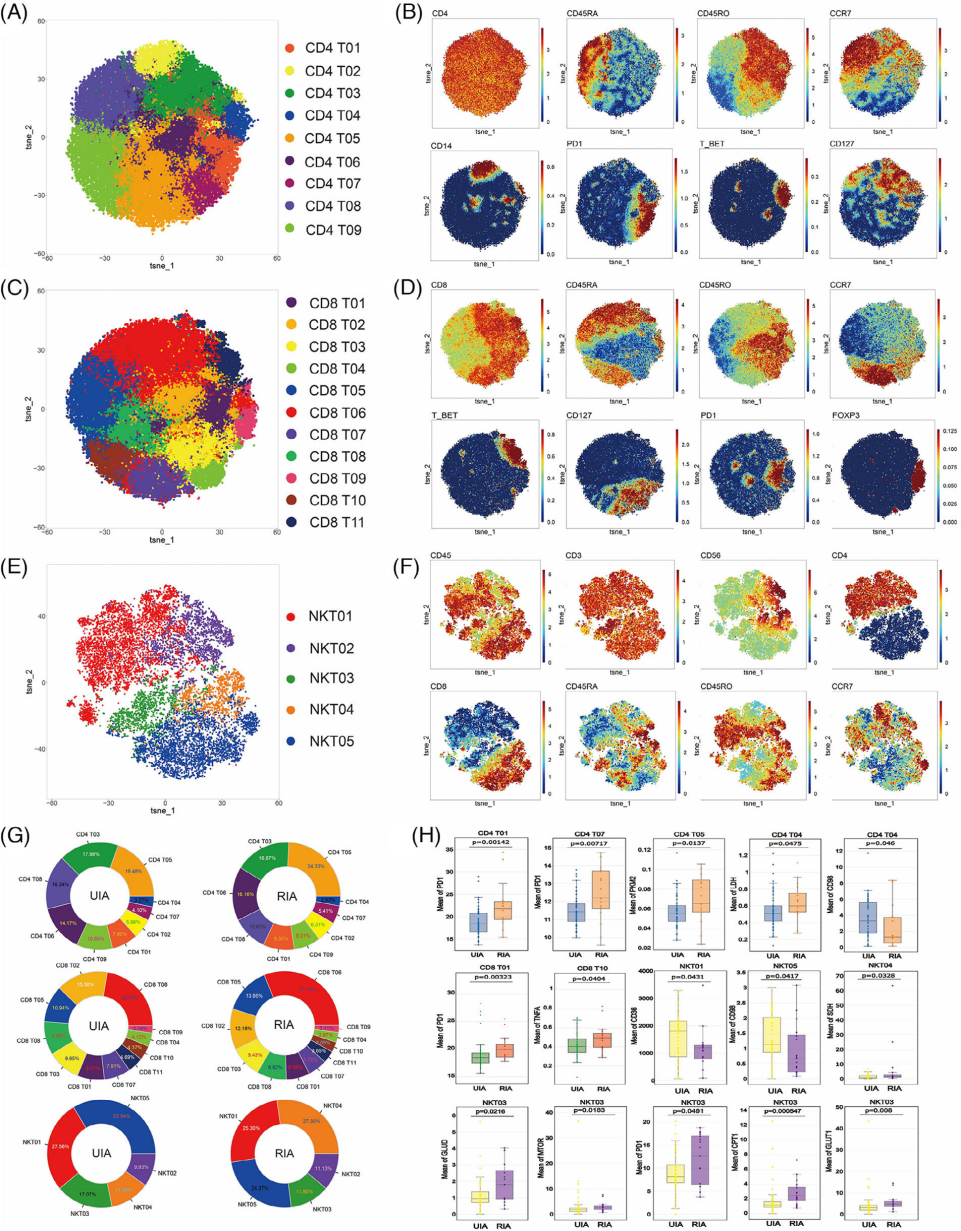
Atlas of T cells (A–G): The results of clustering analysis about CD4 T cells (A), CD8 T cells (C), NKT cells (E) are visualized in the T‐SNE plot, with different colors representing distinct cell subpopulations. Classic markers distinguishing CD4 subsets (B), CD8 subsets (D), NKT subsets (F) are displayed in a spectral format on the T‐SNE plot. The doughnut charts (from top to bottom) respectively show the proportions of each subset within CD4 T cells (Top), CD8 T cells (Middle), and NKT cells (Bottom) (G). Comparative analysis of T subsets (H): The Box plots with scatter points show the positive findings of functional and metabolic molecules for each T‐cell subset compared between the two groups (each color block represents a different CD4 T cell subpopulation, fill colors correspond to the T‐SNE, arranged counterclockwise from high to low starting at 3 o'clock). The different fill colors represent the comparison results from different T cell subsets (CD4 T, CD8 T, or NKT). Detailed comparison results are available in Figure S4–S6.

To explore the differences in T cells between the UIA and RIA groups, we isolated T cell subtypes (CD4 T, CD8 T, and NKT) for Flowsom clustering analysis. The results revealed nine subgroups of CD4 T cells (Figure 1A), 11 subgroups of CD8 T cells (Figure 1C), and five subgroups of NKT cells (Figure 1E). Based on the expression patterns of CD45RA, CD45RO, and CCR7 (Figure 1B,D), CD4 T09 and CD8 T10 were considered Tn cells; five groups of CD4 T cells (CD4 T01, CD4 T02, CD4 T03, CD4 T04, and CD4 T06) and one group of CD8 T cells (CD8 T04) was identified as central memory T cells (Tcm); two groups of CD4 T cells (CD4 T05 and CD4 T07) and five groups of CD8 T cells (CD8 T01, CD8 T02, CD8 T03, CD8 T08, and CD8 T09) were designated as effector memory T cells (Tem). CD8 T05 and CD8 T06 were considered terminally differentiated effector memory cells (Temra). Among them, CD4 T01, CD8 T01, and CD4 T07 belonged to the PD1+ subgroup (Figure 1B,D). Additionally, based on the expression of CD25 and CD56 (Figure S3A), CD8 T04 was identified as a group of effector T cell (Teff). According to Foxp3 expression (Figure 1D), CD8 T09 was considered a group of regulatory T cells (Treg). For NK cells, they were broadly categorized into CD4+ T cell subgroups (NKT01 and NKT02), CD8+ T cell subgroups (NKT04 and NKT05), and double negative T cell (DN‐NKT) subgroup (NK03) based on the expression patterns of CD4 and CD8 (Figure 1F). Among these, NKT02 and NKT04 belong to the high expression of CD56 subgroup, whereas other cells belong to the low expression subgroup (Figure 1F).

Whether in the UIA group or the RIA group, CD4 T05 (UIA: 19.48% and RIA: 24.33%) and CD8 T06 (UIA: 22.34% and RIA: 31.14%) were respectively the most abundant CD4 T (Figure 1G, top) and CD8 T cell subsets (Figure 1G, middle) in peripheral. Among NKT cells, NKT05 (33.54%) was the most abundant subset in the UIA group, whereas NKT04 (27.30%) was the most abundant in the RIA group (Figure 1G, bottom). Comparing the proportions of various T cell subsets between the two groups, we found that the RIA group has more PD1+ CD4 Tem (CD4 T07) (*p* = 0.00406 < 0.01), whereas the UIA group has more transitional memory subset (CD4 T08) (*p* = 0.0108 < 0.05) (Figure S3B–D).

Metabolic molecule comparative analysis revealed that CD56^dim^ DN‐NKT (NKT03) was the subset with the greatest difference among T cells (Figure 1H). In the RIA group, NKT03 showed upregulated expression of GLUD (*p* = 0.0216 < 0.05), GLUT1 (*p* = 0.008 < 0.05), mTOR (*p* = 0.0183 < 0.05), and CPT1 (*p* = 0.000547 < 0.01). The CD4 Tcm (CD4 T04) exhibited upregulated LDH (*p* = 0.0475 < 0.05) in the RIA group, whereas CD98 (*p* = 0.046 < 0.05) was upregulated in the UIA group (Figure 1H). Furthermore, the RIA group showed upregulated PKM2 (*p* = 0.0137 < 0.05) in the CD4 Tem (CD4 T05) and upregulated SDH (*p* = 0.0328 < 0.05) in the CD56^bright^ CD8+ NK (NKT04) subset (Figure 1H). In addition, functional molecule comparisons revealed upregulated expression of the PD1 molecule in several T cell subsets in the RIA group (CD4 T01, CD4 T07, CD8 T01, and NKT03) (Figure 1H).

### 2.2 GATA3+ B cells from RIA exhibited downregulation of PKM2

**FIGURE 2:**
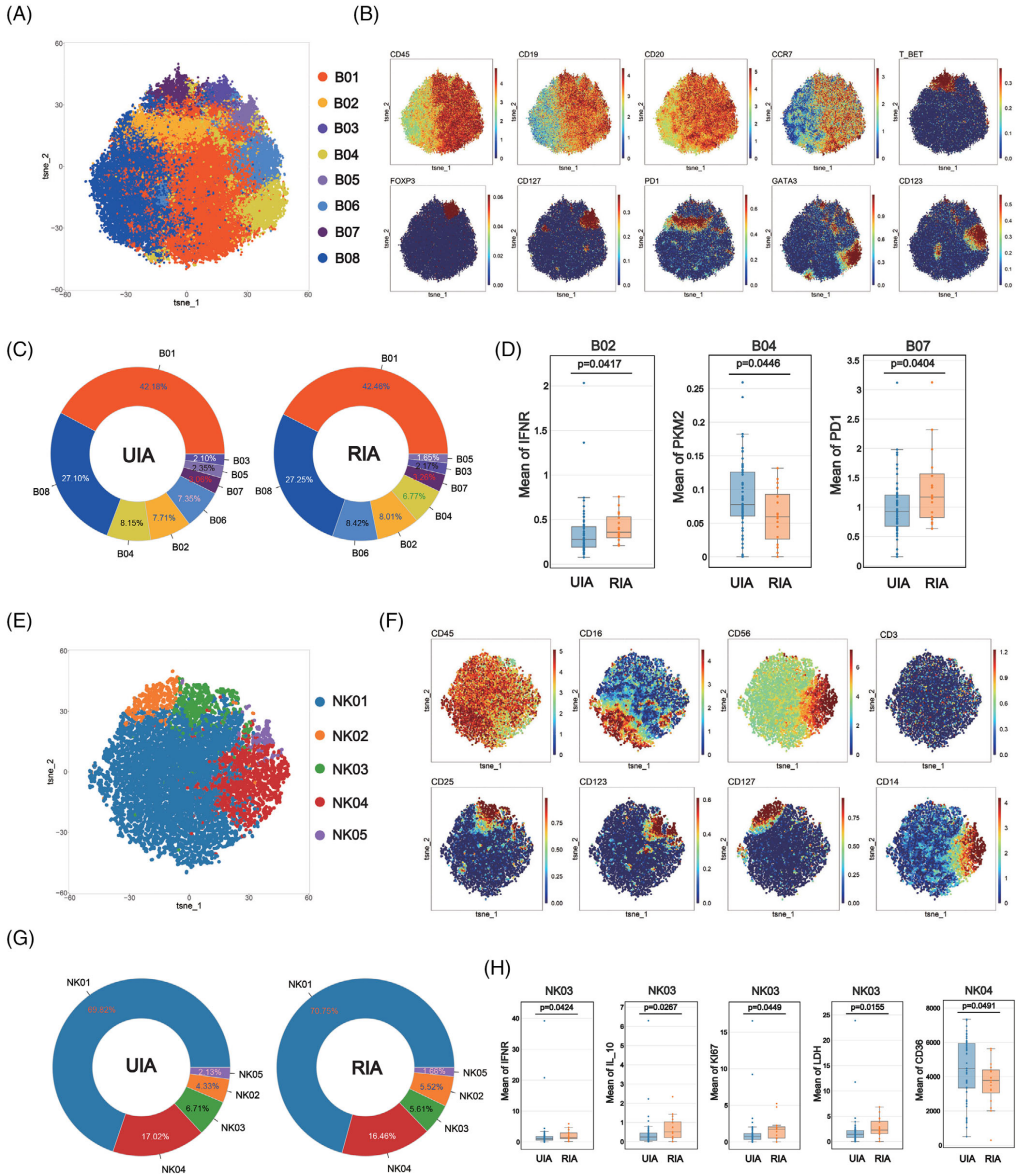
Atlas of B cells (A–C) and NK cells (E‐G): The results of clustering analysis about B cell (A) and NK cells (E) are visualized in the T‐SNE plot, with different colors representing distinct cell subpopulations. Classic markers distinguishing B cells (B) and NK cells (F) are displayed in a spectral format on the T‐SNE plot. The doughnut charts show the proportions of each subset within B cells (C) and NK cells (G). Comparative analysis of B subsets (D) and NK subsets (H): The Box plots with scatter points show the positive findings of functional and metabolic molecules for each B subsets (D) and NK subsets (H) compared between the two groups. Detailed comparison results are available in Figure S7–S8.

To explore potential differences in B cells between RIA and UIA, B cells were isolated and subjected to Flowsom clustering analysis. A total of eight distinct cell subsets were identified based on the expression of surface molecules (Figure 2A). Among them, B08 was classified as a CD19−CD20+ cell subgroup, whereas the other subgroups belonged to the CD19+CD20+ category (Figure 2B). Further subdivision of B cells was achieved using additional cell surface markers (Figure 2B).

Comparing the proportions of various B cell subsets between the two groups, it was observed that B01 was the most abundant subgroup in both UIA (42.18%) and RIA (42.46%) (Figure 2C). The least cell subset in the UIA group was B03 (2.10%), whereas B05 held that position in the RIA group (1.65%) (Figure 2C). Comparative analysis between the two groups did not reveal any statistically significant differences (Figure S3E).

Comparative analysis of metabolism molecules among various cell subsets between the two groups revealed that GATA3+ B cells (B04) from the UIA group expressed higher levels of the PKM2 compared with the counterpart in RIA group (*p* = 0.0446 < 0.05) (Figure 2D). The functional molecule comparison also revealed that PD1+ B cells (B02) and T‐bet+ B cells (B07) from RIA respectively showed higher expressions of IFNγ (*p* = 0.0417 < 0.05) and PD1 (*p* = 0.0404 < 0.05) (Figure 2D).

### 2.3 CD56^dim^ NK cells from RIA showed upregulated expression of LDH

To explore differences in NK cells between the UIA and RIA groups, NK cells were isolated and subjected to Flowsom clustering analysis, revealing five distinct subsets (Figure 2E). Based on the expression of CD56 molecule, NK04 and NK05 belonged to the CD56^bright^ subgroup, whereas the remaining cells were categorized as CD56^dim^ subgroup (Figure 2F).

The proportions of various NK subsets were similar between the two groups, with NK01 constituting the majority (UIA: 69.82%; RIA: 70.75%) and NK05 being the least represented (UIA: 2.13%; RIA: 1.66%) (Figure 2G). Comparative analysis between the two groups did not reveal any statistically significant differences (Figure S3F).

Comparative analysis of metabolic profiles of NK subsets between the two groups revealed that CD56^dim^ NK (NK03) from the RIA group expressed higher levels of the LDH (*p* = 0.0155 < 0.05) and Ki67 (*p* = 0.0449 < 0.05) (Figure 2H). CD14+CD56^bright^ NK (NK04) from the UIA group displayed increased expression of CD36 (*p* = 0.0491 < 0.05) (Figure 2H). Functional molecule comparison revealed that NK03 from the RIA group exhibited high expression of IFNγ (*p* = 0.0424 < 0.05) and IL10 (*p* = 0.0267 < 0.05) (Figure 2H).

### 2.4 mDC and iMo from RIA showed upregulated expression of LDH and CPT1, whereas ncMo upregulated LDH and PKM2. In addition, a special subset of CD127+ cMo from RIA group upregulated mTOR

**FIGURE 3:**
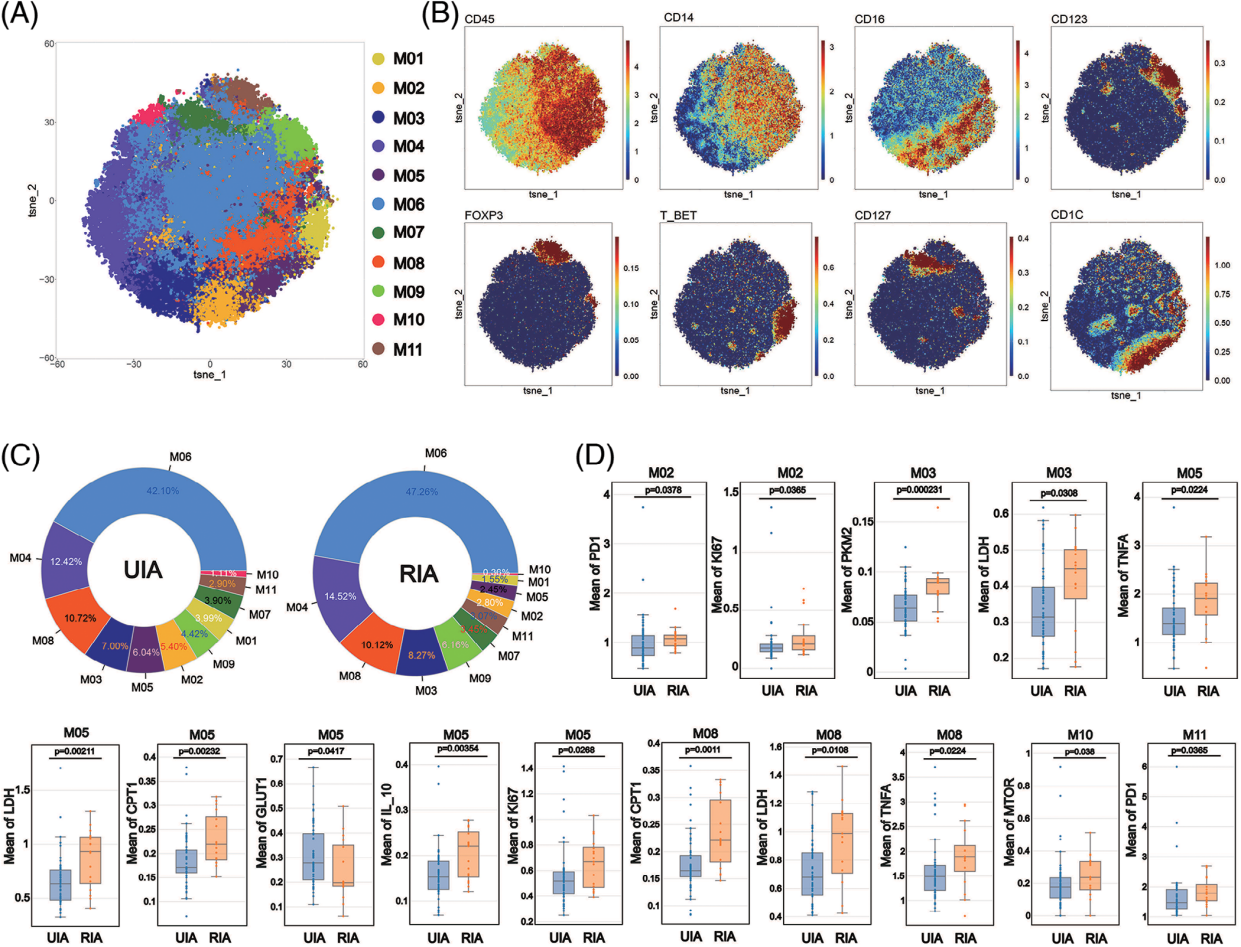
Atlas of monocyte (A–C): The result of clustering analysis about monocyte are visualized in the T‐SNE plot, with different colors representing distinct cell subsets (A). Classic markers distinguishing monocyte are displayed in a spectral format on the T‐SNE plot (B). The doughnut charts show the proportions of each subset within monocyte (C). Comparative analysis of monocyte subsets (D): The Box plots with scatter points show the positive findings of functional and metabolic molecules for each monocyte subsets compared between the two groups. Detailed comparison results are available in Figure S9.

To explore differences in monocytes between the UIA and RIA groups, monocytes were isolated and Flowsom clustering analysis was performed to explore the difference between UIA and RIA groups. The results found 11 distinct subsets (Figure 3A). Based on the expression patterns of CD14 and CD16, the subsets were categorized as CD14+CD16+ intermediate monocytes (iMo) (M01 and M08), CD14−CD16+ nonclassical monocytes (ncMo) (M03) and CD14+CD16− classical monocytes (cMo) (M06, M07, M10, and M11) (Figure 3B). Additionally, M02 and M05 expressed CD1c, resembling two subgroups of myeloid dendritic cells (mDCs) (Figure 3B). M09 expressed CD123, suggesting a potential subgroup of plasmacytoid dendritic cells (pDCs) (Figure 3B).

Comparing the proportions of various monocyte subsets between the two groups, it was observed that M06 represented the most abundant cell subset (UIA: 42.10%; RIA: 47.26%), whereas M10 constituted the least abundant subset (UIA: 1.11%; RIA: 0.36%) (Figure 3C). Comparing the proportions of various monocyte subsets between the two groups, we found that M02 (*p* < 0.001), M05 (*p* < 0.001), and M10 (*p* < 0.001) were enriched in the UIA group, whereas M06 (*p* = 0.00371 < 0.01) and M09 (*p* = 0.0104 < 0.05) were enriched in the RIA group (Figure S3G).

Metabolic molecule comparative analysis revealed that mDC (M05) was the subgroup with the greatest difference among monocytes (Figure 3D). M05 from RIA group showed upregulated expression of LDH (*p* = 0.00211 < 0.01) and CPT1 (*p* = 0.00232 < 0.01), whereas M05 from UIA group showed upregulated expression of GLUT1 (*p* = 0.0417 < 0.05). mTOR was also found to be upregulated in the CD127+ cMo (M10) subset from the RIA group (*p* = 0.038 < 0.05) (Figure 3D). Furthermore, we also observed upregulated expression of metabolic molecules in the ncMo (M03) and iMo (M08) subsets from the RIA group (Figure 3D). Specifically, M03 showed an upregulation of LDH (*p* = 0.0308 < 0.05) and PKM2 (*p* = 0.000231 < 0.01), whereas M08 exhibited an upregulation of LDH (*p* = 0.0108 < 0.05) and CPT1 (*p* = 0.0011 < 0.01) (Figure 3D). Functional molecule comparisons revealed upregulation of the PD1 molecule in the M02 (*p* = 0.0378 < 0.05) and M11 (*p* = 0.0365 < 0.05) from RIA group (Figure 3D). Higher expression of TNF‐α was also observed in M05 (*p* = 0.0224 < 0.05) and M08 (*p* = 0.0224 < 0.05) with significantly different metabolic markers (Figure 3D).

### 2.5 CD101+HLADR+ neutrophils were enriched in the RIA group and various subsets from RIA group downregulation of anti‐inflammatory factors

**FIGURE 4:**
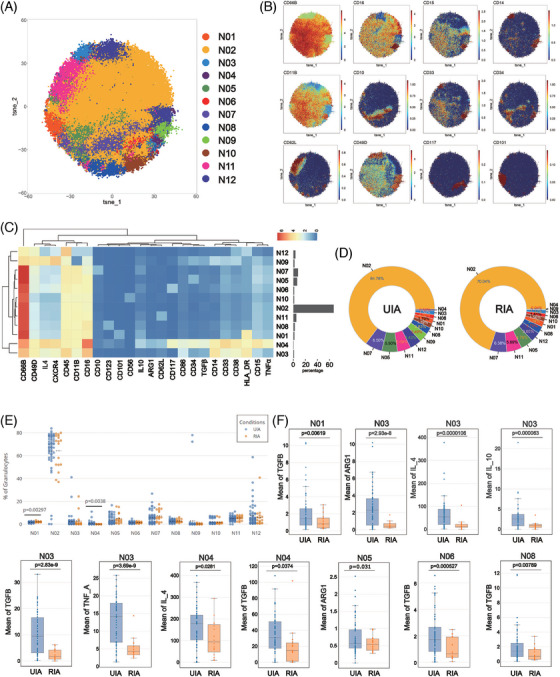
Atlas of neutrophil (A–E): The T‐SNE plot (A) illustrates the results of neutrophil clustering analysis, with distinct subpopulations color‐coded. Classic markers distinguishing these subpopulations are presented in a spectral format on the T‐SNE plot (B). A heatmap (C) depicts the surface marker expression patterns of various cell subpopulations. Two donut plots provide insight into the distribution of neutrophil subpopulations within the UIA and RIA groups (each color block represents a distinct NK subpopulation, with fill colors corresponding to the T‐SNE plot, arranged counterclockwise from high to low starting at 3 o'clock) (D). Comparative analysis of neutrophil subsets (E and F): Scatter plots illustrate the relative proportions of each neutrophil subpopulation between the two groups (Statistically significant differences are denoted by labeled p‐values) (E). Box plots with scatter points unveil significant findings when comparing functional and metabolic molecules between the two groups (F) (For a comprehensive overview of the comparison results, please refer to Figure S10).

To explore differences in neutrophils between the UIA and RIA groups, isolated granulocytes were analyzed using a particular antibody panel. The Flowsom clustering was employed to investigate subsets, revealing a total of 12 cell clusters (Figure 4A). Among these, N03, N04, and N09 expressed CD33 and CD14, resembling polymorphonuclear myeloid‐derived suppressor cells (PMN‐MDSCs) (Figure 4B,C). N01 and N12 exhibited HLA‐DR expression, suggesting distinctive functional statuses. N05, N06, and N08 respectively showed CD34, CD117, and CD123 expression, implying their association with immature subsets (Figure 4B,C). Conversely, mature markers CD10 and CD101 were respectively detected on N07 and N01 surfaces, characterizing these clusters as mature subsets. N02 exhibited typical neutrophil surface markers (CD66b+CD11b+CD15+CD14−CD16+) (Figure 4B,C). Additionally, N10 showed CD56 expression, delineating distinct cellular subset (Figure 4B,C).

Regardless of the RIA or UIA groups, N02 constituted the majority of the cell subset (UIA: 64.76%; RIA: 70.04%). Meanwhile, N04 represented the least abundant subset in both groups (UIA: 0.56%; RIA: 0.04%) (Figure 4D). Comparative analysis of subset proportions between the two groups revealed N01 was predominantly observed in the RIA group (*p* = 0.00297 < 0.01), whereas N04 exhibited higher occurrence in the UIA group (*p* = 0.0038 < 0.01) (Figure 4E).

Functional analysis revealed a more active state in the UIA group. N03 exhibited the most significant functional differences between two groups, with all functional molecules expressed at higher levels in UIA group (Figure 4F). N04 from the UIA group expressed higher levels of IL4 (*p* = 0.0281 < 0.05) and TGFβ (*p* = 0.0374 < 0.05) compared with the RIA group. N05 from the UIA group exhibited elevated expression of ARG1 (*p* = 0.031 < 0.05) (Figure 4F). Additionally, elevated TGFβ expression was observed in N01 (*p* = 0.00619 < 0.01), N06 (*p* = 0.000527 < 0.001), and N08 (*p* = 0.00789 < 0.01) from the UIA group (Figure 4F).

### 2.6 Distinct immune cell interactions between UIA and RIA group

To investigate differences in the associations between immune cell subsets in UIA and RIA groups, we performed correlation analyses on the proportions of major immune cell subsets discovered by cyTOF (including major cell types, high and low metabolic subsets, clusters with substantial metabolic differences).

**FIGURE 5:**
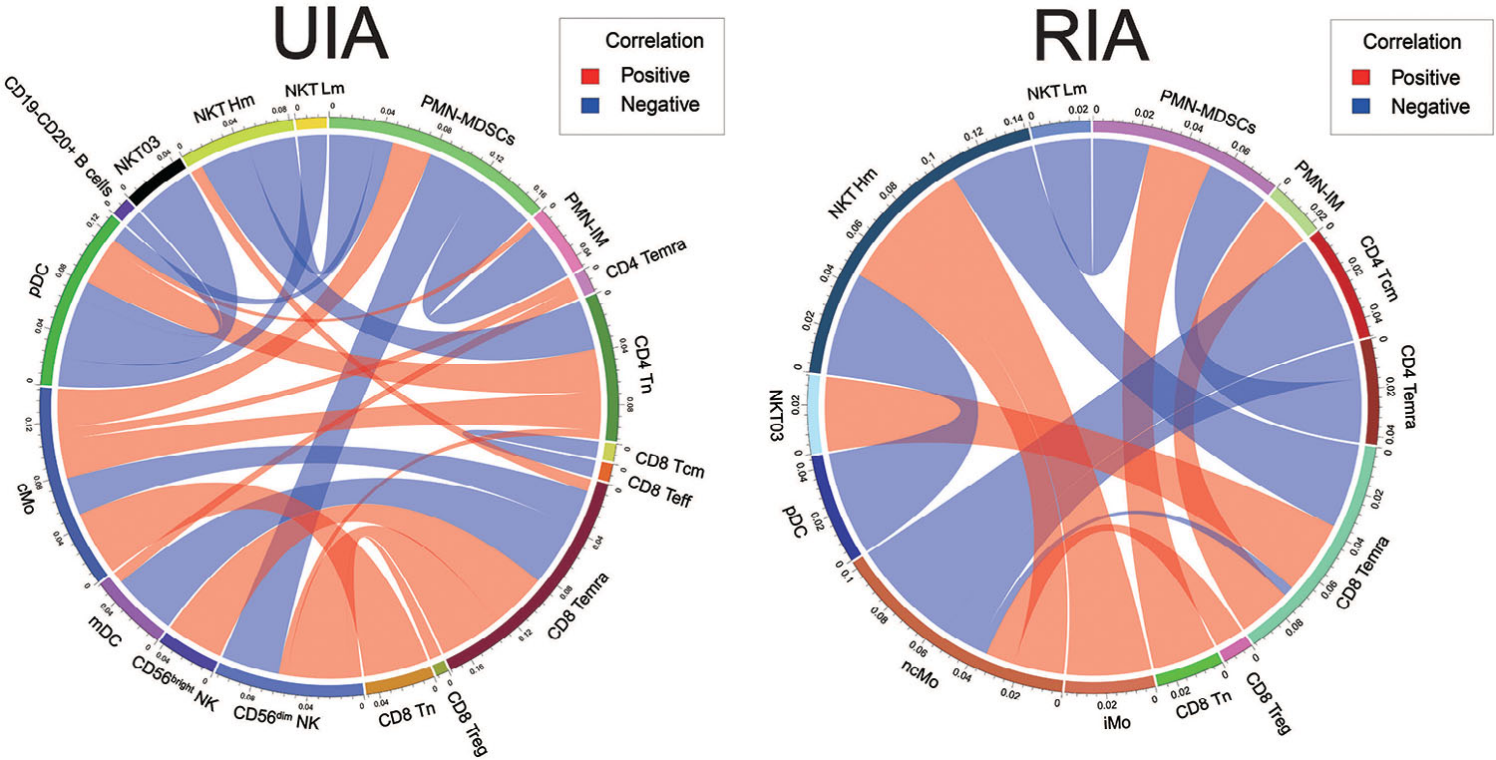
Peripheral blood immune cell correlation analysis (A): The Circos plot illustrates the statistically significant correlations between major cell subsets in peripheral blood of UIA and RIA group (red lines represent positive correlations, while blue lines represent negative correlations).

The statistically significant correlation results showed that the associations among cells were more complex and diverse in the UIA group. Specifically, in the UIA group, the correlations were mainly associated with pDCs, CD56^dim^ NK, CD8 Temra, and cMo. CD56^dim^ NK were positively correlated with CD8 Treg, CD8 Temra, and CD4 Tn, whereas negatively correlated with PMN‐MDSCs (N03, N04, and N09). CD8 Temra were positively correlated with CD56^bright^ NK and NKT Hm (high metabolic subsets, NKT02 and NKT04), and negatively correlated with mDCs and cMo. pDCs were positively correlated with CD4 Tn, PMN‐IM (immature PMNs, N05, N06, and N08), and negatively correlated with PMN‐MDSCs, NKT Lm (low metabolic subsets within NKT, excluding NKT02 and NKT04), NKT03 (clusters with highly divergent metabolic profiles), and CD19−CD20+ B cells. cMo were positively correlated with CD4 Tn, CD4 Temra, and PMN‐MDSCs. Additionally, we observed negative correlations between NKT Hm and PMN‐MDSCs and between CD8 Tcm and CD8 Teff. PMN‐IM showed negative correlations with PMN‐MDSCs (Figure 5, left).

In the RIA group, the correlations mainly involved ncMo, CD8 Temra, and NKT Hm. Specifically, CD8 Treg and NKT Hm exhibited positive correlations with ncMo, whereas CD8 Temra, CD4 Temra, and CD4 Tcm showed negative correlations. CD8 Temra were positively correlated with PMN‐IM and NKT03 but negatively correlated with NKT Hm. NKT Hm showed negative correlations with pDCs and positive correlations with iMo. Furthermore, PMN‐MDSCs displayed negative correlations with CD4 Temra and NKT Lm but a positive correlation with CD8 Tn (Figure 5, right).

### 2.7 RNA‐seq analysis of intracranial aneurysm tissues

**FIGURE 6:**
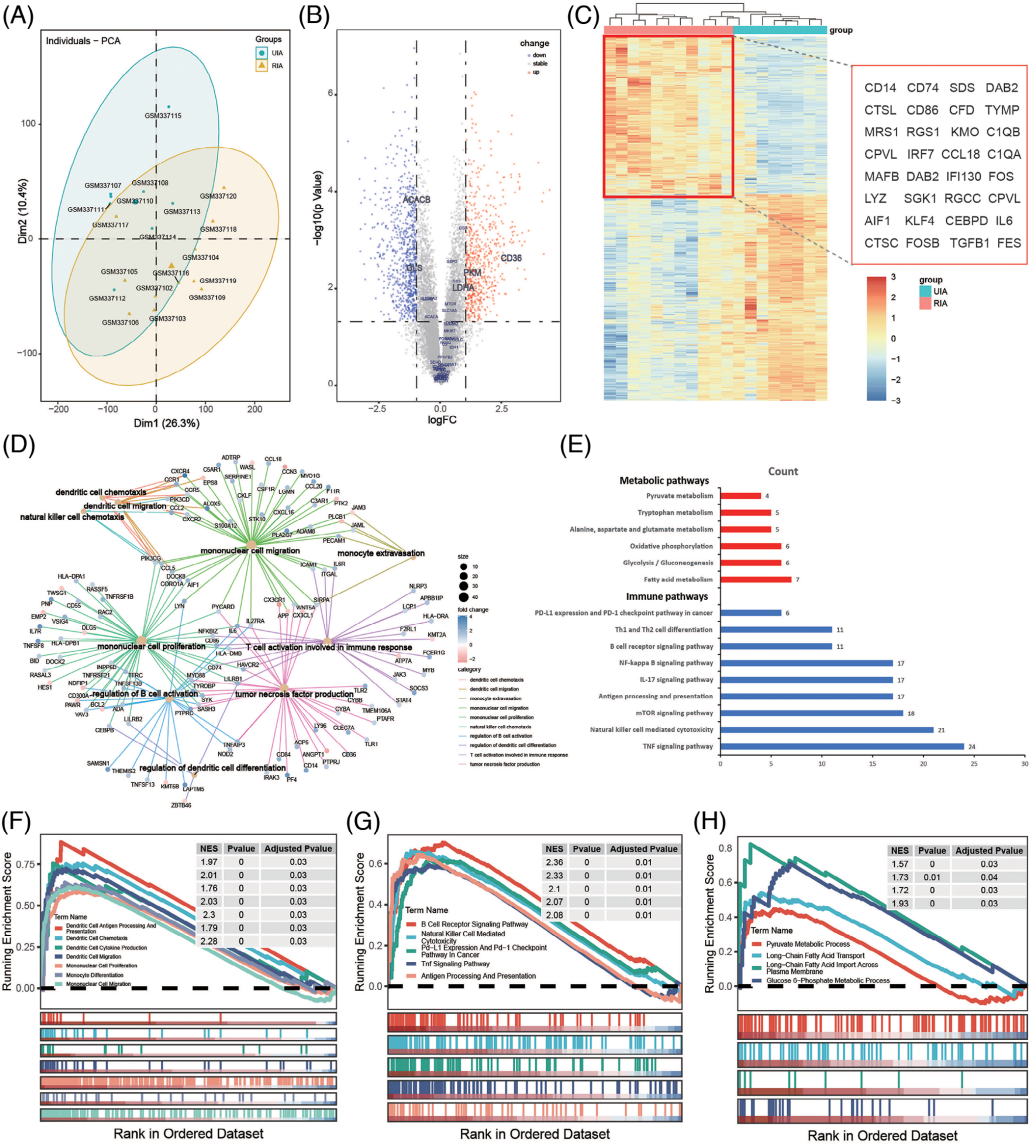
Comparative analysis of RNA‐seq data from 11 UIA and 8 RIA tissues. PCA shows the gene expression patterns of each sample between the two groups (A). The volcano plot displays the differentially expressed genes between UIA and RIA, with blue font highlighting metabolism‐related genes (B). The heatmap shows the distribution of differentially expressed genes between the two groups, with a red box indicating the expression of genes associated with monocytes and mDC cells (C). The petal plot illustrates the GO enrichment analysis results of differentially expressed genes associated with monocytes and mDC cells (D). Bar charts respectively show the KEEG analysis results of differentially expressed genes in metabolic (red) and immune‐related pathways (blue) (E). GSEA analysis results of differentially expressed genes associated with monocytes and mDC cells (F), other immune cells (G), and metabolism (H).

To further analyze the metabolic changes of immune cells in intracranial aneurysm tissues, we analyzed RNA‐seq data from 11 RIA tissues and 8 UIA tissues from the GEO database (Figure 6A). GO and KEGG enrichment analysis revealed that differentially expressed genes (DEGs) were mainly involved in immune processes related to monocytes and DC cells (Figure 6C). Additionally, DEGs were also enriched in immune processes such as T cell activation, B cell activation regulation, and tumor necrosis factor production (Figure 6D). These results were consistent with the findings of CyTOF analysis, highlighting the roles of monocytes, DCs, T cells, and B cells in the rupture of intracranial aneurysms. Furthermore, we focused on the differential expression of genes related to cell metabolism between the two groups. The results showed that metabolism‐related genes were mainly enriched in pathways such as fatty acid metabolism, glycolysis/gluconeogenesis, and oxidative phosphorylation (Figure 6E). Additionally, DEGs were also found in the pathways of tryptophan metabolism, as well as in the metabolism pathways of alanine, aspartate, and glutamate (Figure 6E). Further Gene Set Enrichment Analysis (GSEA) confirmed the upregulation of these DEGs in the RIA group (Figure 6F,G,H).

**FIGURE 7:**
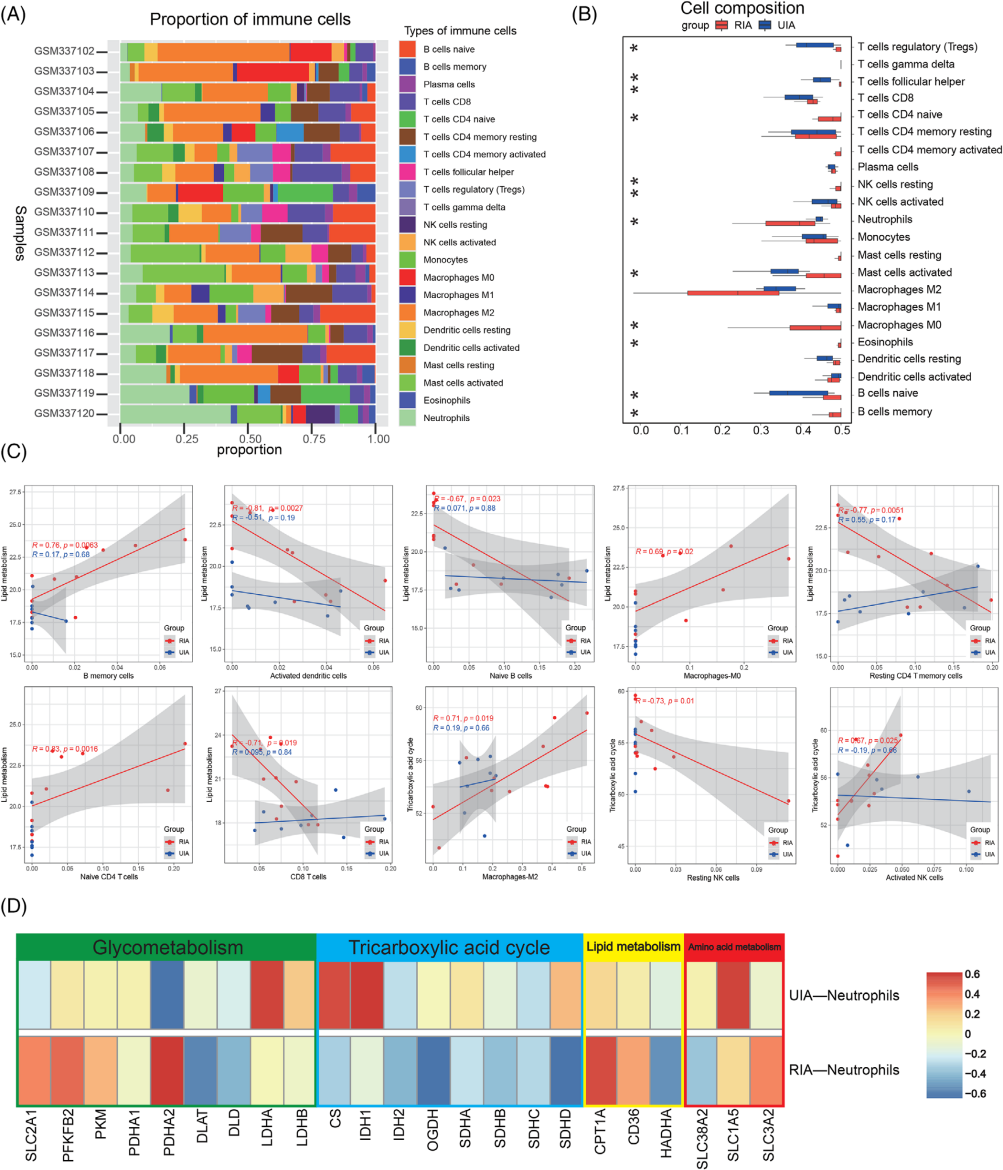
Immune infiltration analysis of RNA‐seq data. The analysis uses the 22 common immune cell gene sets provided by CIBERSORT. Stacked bar plots show the composition of the 22 immune cells in the samples (A). Box plots compare the proportions of the 22 immune cells between RIA and UIA tissues (B). Correlation scatter plot display the correlation between immune cell infiltration scores and metabolism‐related genes in different sample groups (only positive results are shown, all the results can be found in the Figure S11–S14) (C). Heatmaps demonstrate the correlation between metabolism‐related genes (glycolysis, lipid metabolism, TAC cycle, and amino acid metabolism) and neutrophil from UIA and RIA groups (D).

To further analyze the metabolic changes of immune cells in RIA tissues, we conducted immune infiltration analysis of RNA‐seq data. First, the CIBERSORT algorithm was used to assess the composition of immune cells in tissues (Figure 7A). The results showed that the UIA group contained more Tregs, Follicular helper T cell (Tfh), B naive cells, and activated mast cells, whereas the RIA group contained more B memory cells, neutrophils, eosinophils, naive CD4 T cells, M0 macrophages, and resting NK cells (Figure 7B). We then analyzed the correlation between immune cells and common metabolic processes. The statistically significant differences were mainly from the RIA group. Among them, lipid metabolism‐related genes were positively correlated with B memory cells (*p* = 0.0063 < 0.05, *R* = 0.76), M0 macrophages (*p* = 0.02, *R* = 0.69), and CD4 T naive cells (*p* = 0.0016 < 0.05, *R* = 0.83) from the RIA group, whereas negatively correlated with activated DC cells (*p* = 0.0027 < 0.05, *R* = −0.81), B naive cells (*p* = 0.023 < 0.05, *R* = −0.67), resting CD4 T memory cells (*p* = 0.0051 < 0.05, *R* = −0.77), and CD8 T cells (*p* = 0.019 < 0.05, *R* = −0.71) from the RIA group (Figure 7C). Furthermore, we found that tricarboxylic acid cycle (TAC) cycle‐related genes were negatively correlated with resting NK cells (*p* = 0.01 < 0.05, *R* = −0.73) from the RIA group, whereas positively correlated with activated NK cells (*p* = 0.025 < 0.05, *R* = 0.67) from the RIA group (Figure 7C). Similarly, M2 macrophages (*p* = 0.019 < 0.05, *R* = 0.71) from the RIA group were positively correlated with TAC cycle‐related metabolic genes (Figure 7C). We also compared the metabolic profiles of neutrophils from the RIA and UIA groups. The results showed that TAC cycle‐related genes were positively correlated with neutrophils from the UIA group and negatively correlated with neutrophils from the RIA group. To be specific, a comparison revealed that neutrophils from the RIA group were negatively correlated with OGDH (*p* = 0.025 < 0.05) (Figure 7D). In addition, the glucose metabolism‐related gene PDHA2 was negatively correlated with neutrophils from the UIA group (*p* = 0.037 < 0.05) and positively correlated with neutrophils from the RIA group (*p* = 0.037 < 0.05) (Figure 7D).

These results suggests that there are differences between the local environment and the peripheral immune environment. When peripheral immune cells infiltrate local lesions, corresponding metabolic reprogramming may occur, altering their functions and thus affecting the stability of aneurysms.

## 3 DISCUSSION

IA is a potentially fatal cerebrovascular disease, typically forming at bifurcation points or curved segments of intracranial arteries.^25^, ^26^ Although most IAs are asymptomatic, their rupture can lead to SAH, which is an acute life‐threatening cerebrovascular event.^27^, ^28^ Changes in the peripheral immune environment play an important role in the rupture and prognosis of intracranial aneurysms.^29^ This study utilized single‐cell CyTOF technology to explore the peripheral immune composition and changes after rupture from the perspectives of metabolic alterations and heterogeneity. These findings contribute to a better understanding of the underlying mechanisms of peripheral immune environment changes after intracranial aneurysm rupture, providing new insights for disease prevention and treatment.

PBMCs are the main builders of the peripheral immune environment, and their functional changes are mainly regulated by their own metabolic mode.^12^ Immune cells required amount of energy to carry out various tasks when responding to infections or inflammation.^11^ Metabolism reprogramming ensures that immune cells can efficiently perform their functions.^30^, ^31^ In our study, metabolic analysis of PBMCs uncovered unique metabolic traits within different subgroups. Overall, most PBMCs subsets from RIA group showed upregulation of rate‐limiting enzymes related to the glycolytic pathway. However, some subsets of PBMCs in UIA group mainly showed upregulation of amino acid and lipid uptake. PKM2 and LDH, play pivotal roles in the cellular glycolysis pathway. Studies have shown that the activation of the glycolytic pathway is closely associated with the inflammatory activation of immune cells.^32^, ^33^ In certain diseases, PKM2 and LDH emerge as pivotal molecular determinants regulating pro‐inflammatory metabolic adaptation.^34^, ^35^ T‐bet is a typical marker of Th1 cells. Th1 plays an important role in the peripheral immune response due to its potential to release proinflammatory factors and participate in the activation of a variety of immune cell functions.^36^ Glycolytic transition may mediate the activation of T‐bet+ CD4 Tcm, and then participate in the peripheral mechanism of the rupture. In addition, the glycolytic upregulation was also observed in multiple monocytes and NK subsets. ncMo and iMo are often classified into tissue‐associated monocytes.^37^ Among them, iMo and ncMo mainly play a role in antigen presentation and immune regulation. Studies have shown that their activation can produce a variety of proinflammatory cytokines, so they are often considered as a subset of inflammatory monocytes.^38^, ^39^ The function of CD56^dim^NK is produced by releasing cytotoxic particles and expressing receptors that regulate cell apoptosis. CD56^dim^NK cells are generally more cytotoxic and have a wider range of effector functions than CD56^bright^NK cells.^40^ Glycolytic metabolic transition is closely related to the exercise of peripheral functions. This can also be seen from the upregulation of iMo and CD56^dim^NK accompanied by the proinflammatory factor TNF‐α. CD56^dim^ DN‐NKT and CD25+ mDC were the cell subsets with the most differences in metabolic markers between the two groups. Among them, in addition to the upregulation of glycolysis‐related markers, we also observed the upregulation of CPT1, which mediates the transport of long‐chain fatty acids into mitochondria. Similarly, in the RNA‐seq analysis of aneurysm tissue, the degree of activated NK cell infiltration was positively correlated with the expression of core genes about TAC cycle, indicating the active metabolic capabilities of NK cells within the local lesion. This phenomenon suggests that NK cells play an important role in mediating immune dysregulation within the local lesion and in mediating the rupture of aneurysm. Studies have shown that the differentiation of peripheral blood CD1c+ mDC could directly affect Th1/Th2 balance.^41^ CD56^dim^ DN‐NKT may be a cytotoxic subset similar to CD56^dim^ NK. As a rate‐limiting enzyme in fatty acid β‐oxidation, the upregulation of CPT1 in RIA group suggests that the pattern shift in lipid metabolism regulated the function of these immune cells. In the RNA‐seq analysis of aneurysm tissue, we also found a correlation between lipid metabolism genes and immune infiltration scores. This further indicate that lipid metabolism regulation also played an important role in regulating the function of immune cells within the local lesion of intracranial aneurysms. Interestingly, we found that GATA3+ B cells from the RIA group exhibited a metabolic state with low PKM2 expression. Studies have found that GATA3 is mainly involved in inhibiting the expression of B cell‐related genes during the early differentiation of lymphocytes.^42^ The enhanced glycolysis of B cell subsets with special phenotypes may be related to their involvement in maintaining the stability of peripheral environment of UIA. High expression of PD1 in immune cells was associated with immunosuppression. However, we observed elevated PD1 expression in various subsets derived from RIA patients, which may be considered an adaptive response to functional hyperactivation.^43^ mTOR is a protein kinase closely associated with protein synthesis and cell proliferation.^44^ B cells have a strong ability to synthesize antibodies, which may be why mTOR was highly expressed. Studies have found that mTOR can promote the functional differentiation of immune cells by regulating cell metabolism, especially glucose metabolism and lipid synthesis.^45^ The higher expression of NKT03 and M10 observed in the RIA group suggests that these cells undergo metabolic reprogramming and activation of functions. These metabolic changes may directly or indirectly regulate the functional activities of these peripheral immune cells, thereby mediating the execution of their respective functions.

Neutrophils have traditionally been considered a homogeneous population. But in recent years, it has been discovered that they contain multiple heterogeneous subsets.^46^, ^47^ Our study also found the presence of heterogeneous neutrophil subsets in the peripheral blood of patients with IAs. These populations can be broadly categorized into PMN‐MDSCs, fully mature neutrophils, and immature neutrophils. In our study, we observed that CD101+HLA_DR+ neutrophils were predominantly present in the peripheral blood of RIA group. CD101 is regarded as a surface marker for mature neutrophils, whereas HLA_DR is typically expressed on neutrophils only under specific inflammatory conditions.^48^, ^49^ Both markers are closely associated with the pro‐inflammatory activation of neutrophils.^50^, ^51^ MDSCs typically play a crucial role in immune regulation, primarily by suppressing the activity of immune cells to maintain immune balance and prevent excessive immune responses.^52^, ^53^ The anti‐inflammatory characteristics of PMN‐MDSCs, which were enriched in the UIA group, contribute to the alleviation of the peripheral immune inflammatory environment. N03 as another subset of MDSCs showed significant functional differences between the two groups. N03 from the UIA group displayed relatively stronger anti‐inflammatory functions compared with the RIA group, with higher expression of various anti‐inflammatory molecules. This suggests that the impaired anti‐inflammatory function of the corresponding subgroups in the UIA group may be involved in the rupture.

In summary, our study investigated the peripheral mechanisms of intracranial aneurysm rupture from the perspectives of PBMCs metabolic changes and neutrophil heterogeneity. These findings provide targets for future precision immunotherapy.

## 4 LIMITATION

This study has the following limitation. Because our study used peripheral blood samples from patients after rupture, although studying patients admitted 6 h after rupture can reduce the impact of confounding factors to some extent, it still cannot avoid the interference of the rupture itself on the causal exploration process. However, from the perspective of single‐cell showing the composition of the peripheral immune environment, this study objectively demonstrates the changes in the peripheral immune environment of patients with ruptured aneurysms. This is beneficial for a deeper understanding of the mechanism of peripheral immune dysfunction in patients after rupture, laying the foundation for further exploration of prognostic analysis after rupture.

## 5 MATERIALS AND METHODS

### 5.1 Human specimens and ethics statements

**FIGURE 8:**
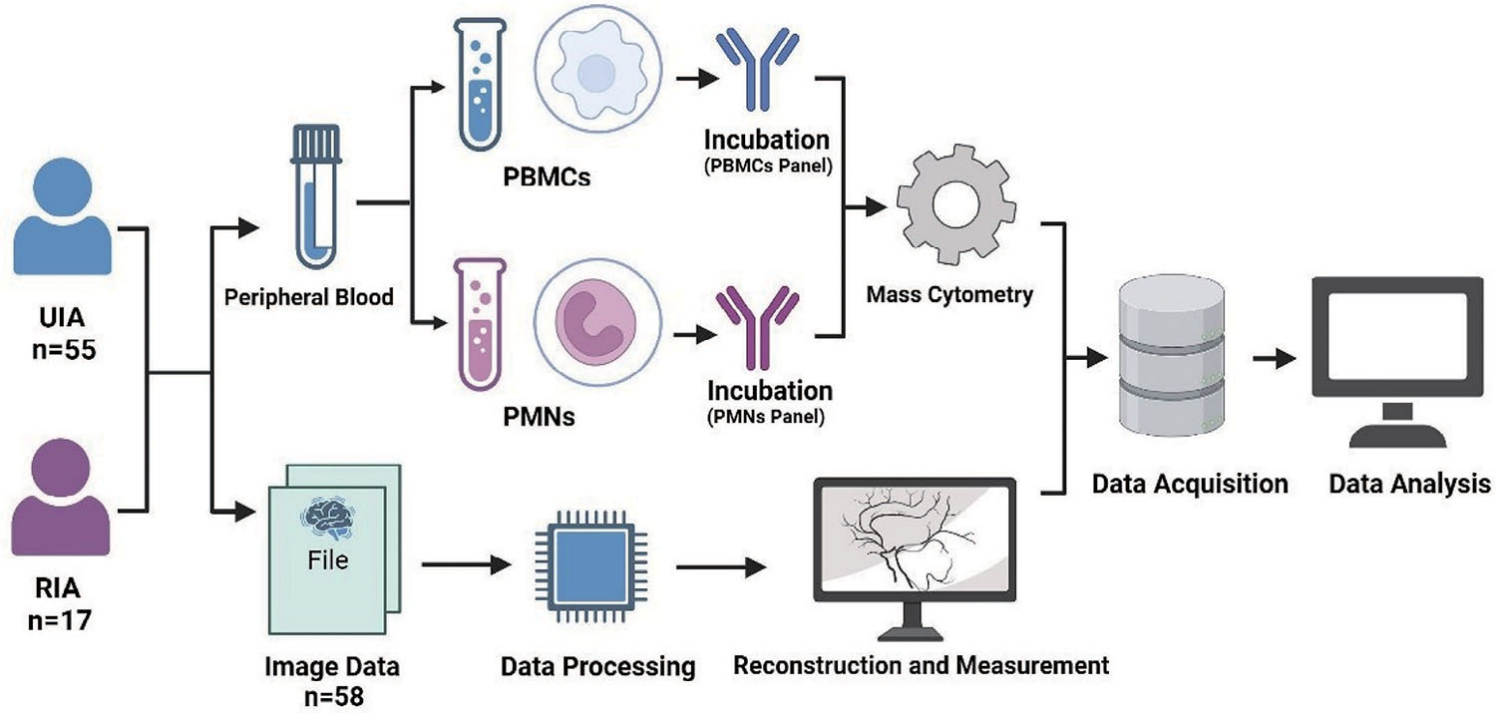
Workflow. 55 patients with unruptured intracranial aneurysms (UIA) and 17 patients with ruptured intracranial aneurysms (RIA) are enrolled in this study. Peripheral blood samples are collected, and PMNs and PBMCs are isolated following centrifugation. After incubation with specific antibodies for PMNs and PBMCs, flow cytometry is performed. Out of the 72 patients, 58 have complete imaging data. These imaging data are extracted, followed by subsequent reconstruction and the measurement of aneurysm morphological parameters. Finally, comprehensive data analysis was conducted.

Between October 2022 and April 2023, 72 cases of IA patients were included according the inclusion criteria (55 cases of UIAs and 17 cases of RIAs). Ruptured aneurysms were determined based on the presence of SAH according to image. *Inclusion criteria*: (a) Patients diagnosed with intracranial aneurysms by digital subtraction angiography, computed tomography angiography (CTA), or magnetic resonance angiography. (b) Patients with ruptured aneurysms who presented symptoms and were admitted within 6 h. *Exclusion criteria*: (a) Patients with tumor diseases; (b) patients with chronic or acute systemic inflammatory diseases; (c) patients using immunosuppressive medications; (d) patients who have received chemotherapy, radiotherapy or treatments potentially impairing the systemic immune system; (e) patients with liver or kidney impairments. All blood samples were collected upon admission. Among them, 58 patients had complete preoperative imaging data. The clinical information of 72 enrolled patients was presented in Table S1 and 58 patients with complete imaging data were presented in Table S2. Figure 8 illustrated the concise workflow of this study.

### 5.2 Isolation and characterization of single cells from PBMCs and PMNs

Fresh blood samples obtained upon admission were collected in EDTA anticoagulation tubes. Within a 2‐h timeframe, the blood samples were overlaid with 5.0 mL of Polymorphprep (Serumwerk Bernburg AG; 1895). Following this, the tubes were centrifuged at 500×*g* for 30 min at a temperature ranging from 18 to 22°C. After centrifugation, the upper band (containing PBMCs) and the lower band (containing PMNs) were carefully aspirated and transferred separately into two centrifuge tubes. These cellular fractions were then resuspended in isotonic saline (0.9% NaCl) and underwent an additional round of centrifugation.

After centrifugation, the cells were washed using RBC lysis buffer (Solarbio; R1010), followed by another round of centrifugation. Subsequently, each tube received 0.5 mL of cisplatin (Fluidigm; 201064) for cell viability labeling. The reaction was halted after a 2‐min incubation by adding 1 mL of 2% FBS (Gibco; 10091−148). Following another centrifugation step and removal of the supernatant, the cells were fixed with 1 mL of 1.5% paraformaldehyde (Biosharp; BL539A) for 15 min. Neutralization was achieved using 2 mL of 2% FBS, followed by another centrifugation step. Finally, the cells were resuspended in a cell preservation solution (Bioteh; C41100) for subsequent analysis.

### 5.3 Mass cytometry

To assess distinctions among PBMCs, we designed a marker set comprising 36 antibodies (markers of distinguishing various PBMC subsets, markers of metabolic profiles, and markers of function). Additionally, to investigate the heterogeneity of PMNs in peripheral blood, we utilized a panel of 24 antibodies (markers of distinguishing PMN subsets and markers of function). These antibodies, purchased in a purified state from BioLegend (San Diego, USA), were subsequently labeled in‐house using the Maxpar X8 Multimetal Labeling Kit (Fluidigm, USA) in strict accordance with the instructions. For a comprehensive list of the antibodies used, along with their corresponding isotopes, please refer to Table S3 (PBMCs panel) and Table S4 (PMNs panel).

### 5.4 CyTOF data analysis

The CyTOF data were obtained in the. fcs file format using the CyTOF2 system. To facilitate normalization, EQ Four Element Beads were incorporate and a MATLAB‐based technique was applied. Subsequently, the data were uploaded to Cytobank (https://premium.cytobank.cn). Initially, bead filtering was conducted, followed by the application of specific gating criteria to isolate single cells. Viable cells were subsequently identified based on 193Ir staining, and CD45+ cells were gated to separate PBMCs and PMNs (refer to Figure S1 for details). Subsequent data analysis was conducted utilizing the automated dimensionality reduction algorithm known as Flowsom, implemented within the R programming environment. Visualization of the outcomes was accomplished through tSNE, a visual dimensionality reduction algorithm.

### 5.5 Vascular reconstruction and measurement of morphological parameters

We obtained preoperative CTA data in DICOM format from a high‐resolution CTA workstation (Siemens, Berlin, Germany) and transformed them into slice DICOM data with a slice thickness of approximately 0.5 mm. Afterward, we imported this dataset into Mimics 19.0 (Mimics Research 19.0; Materialize, Belgium) for subsequent reconstruction and analysis. Radiological assessments were performed by an experienced neurosurgeon (ML) utilizing high‐resolution CTA scans. Measurements of *L*, *d*, *H*, angles (flow angle, aneurysm angle, angle of blood vessel, and mother–child angle), and NSI were derived from the CTA scans, as depicted in Figure S2.

### 5.6 RNA‐seq data analysis

The RNA‐seq data were mainly derived from the GSE13353 dataset in the GEO database, which includes RNA‐seq data from 11 RIAs and eight UIAs (detailed information can be found at https://www.ncbi.nlm.nih.gov/geo/query/acc.cgi?acc=GSE13353). The limma package was used for data normalization. DEGs were selected under the criteria of |log2FoldChange| > 1 and FDR < 0.05. The “org.Hs.eg.db” was used for gene ID conversion, and GO and KEGG enrichment analysis was performed using the “clusterProfiler” R package. Additionally, GSEA analysis of DEGs was conducted using GO and KEGG datasets as references.

A bioinformatics algorithm called CIBERSORT was used to evaluate immune cell infiltrations. The leukocyte gene signature matrix LM22 with 1000 permutations was used to calculate the putative abundance of immune cells (21). The data with a CIBERSORT value of *p* < 0.05 were filtered and retained for the following analysis. Thus, a matrix of immune cells fractions was generated. SLC2A1, PFKFB2, PKM, PDHA1, PDHA2, DLAT, DLD, LDHA, and LDHB were selected as genes of glucose metabolism; IDH1, IDH2, OGDH, SDHA, SDHB, SDHC, and SDHD were selected as genes of the TAC cycle; CPT1A, CD36, and HADHA were selected as genes of lipid metabolism; SLC38A2, SLC1A5, and SLC3A2 were selected as genes of amino acid metabolism. The “ggcorrplot” R package was used to visualize the correlation between these genes and 22 types of immune cells.

### 5.7 Statistical analysis

Continuous clinical variables were presented as mean ± interquartile range and categorical variables were expressed as counts (*n*) and percentages (%). Categorical variables were analyzed using chi‐square tests, normally distributed continuous variables were assessed using *t*‐tests and non‐normally distributed continuous variables were evaluated using Wilcoxon tests. Scatter plots were utilized for visualizing the comparison of subset proportions between the two groups. Intergroup differences were determined using Wilcoxon tests. Following the normalization of subset proportions, Pearson correlation coefficients were computed to examine relationships between different cell subsets. Statistical analyses were performed using R software and significance was defined as a *p* value below 0.05.

## AUTHOR CONTRIBUTIONS

Xiaolong Ya collected blood samples and performed single‐cell suspension extraction. Xiaolong Ya, Chenglong Liu, and Long Ma performed the extraction of PBMCs and PMNs. Peicong Ge gave some advice. Xiaoxue Xu, Zhiyao Zheng, and Siqi Mou helped organize some of the data. Yan Zhang, Rong Wang, Qian Zhang, and Xiaolong Ya provided the guidance for this experiment. Wenjing Wang, Hao Li, and Jizong Zhao supervised this research. All authors have read and approved the final manuscript

## CONFLICT OF INTEREST STATEMENT

The authors declare no conflict of interest.

## ETHIC STATEMENT

This study received approval from the Institutional Review Board (IRB) and Ethics Committee of Beijing Tiantan Hospital (Beijing, China) (KY2017‐035‐02). Written informed consent was obtained from all participating patients and healthy controls.

## Supporting information

Supporting information

## Data Availability

These data were uploaded to Cytobank (https://premium.cytobank.cn). Data can be shared on the platform by contacting us.
