# Supplementary material for: Single‐cell atlas of peripheral blood by CyTOF revealed peripheral blood immune cells metabolic alterations and neutrophil changes in intracranial aneurysm rupture
Source: MedComm (2020). 2024 Jul 15;5(8):e637. doi: 10.1002/mco2.637 (PMC11247334; doi:10.1002/mco2.637)
Supplement: Supplementary file 1 — Supporting information [file MCO2-5-e637-s001.pdf]

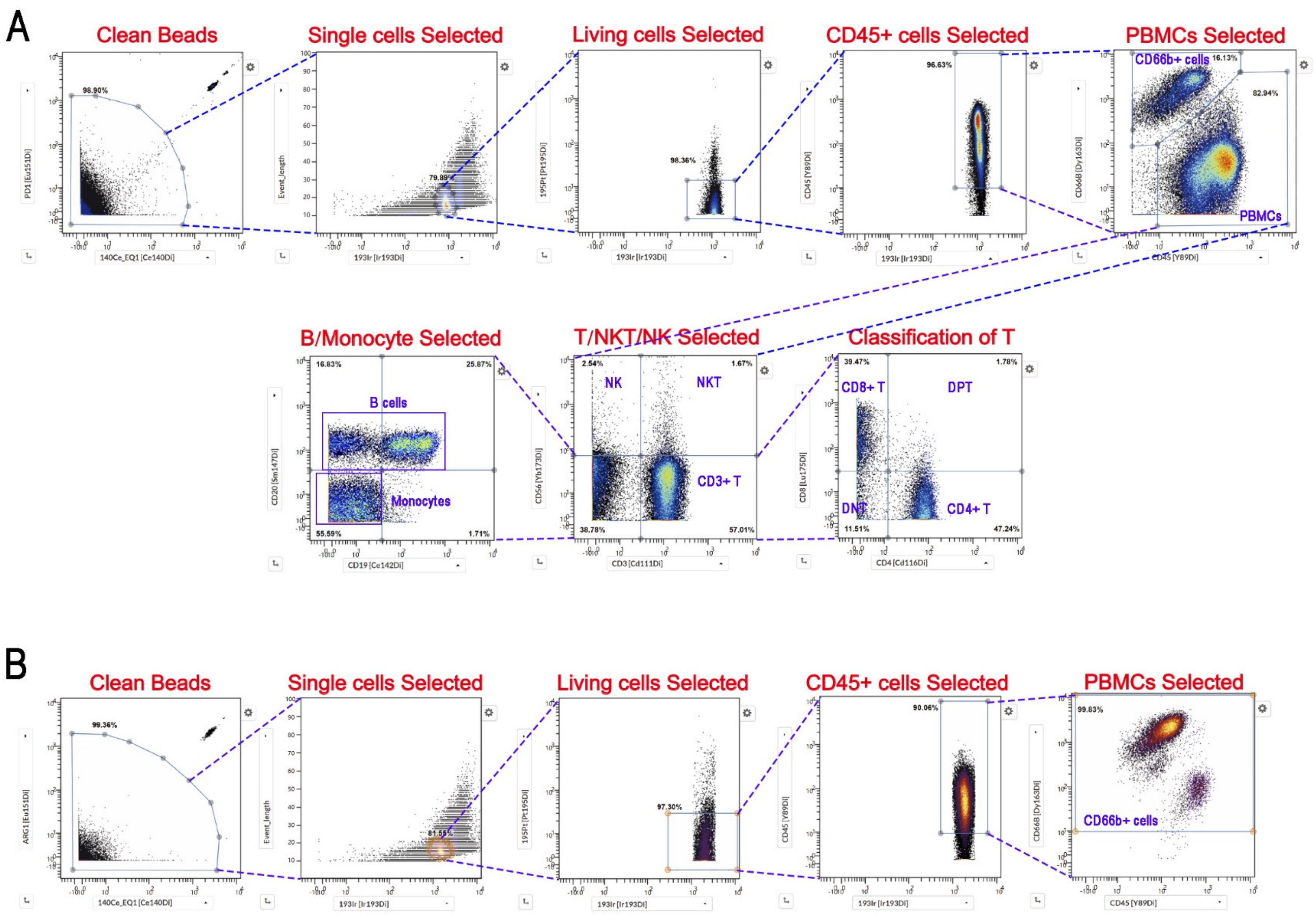

Supplementary Figure 1: CyTOF data preprocessing and cell subset isolation strategies for PBMCs before Flowsom clustering analysis (A). CyTOF data preprocessing and isolation for PMNs (B).

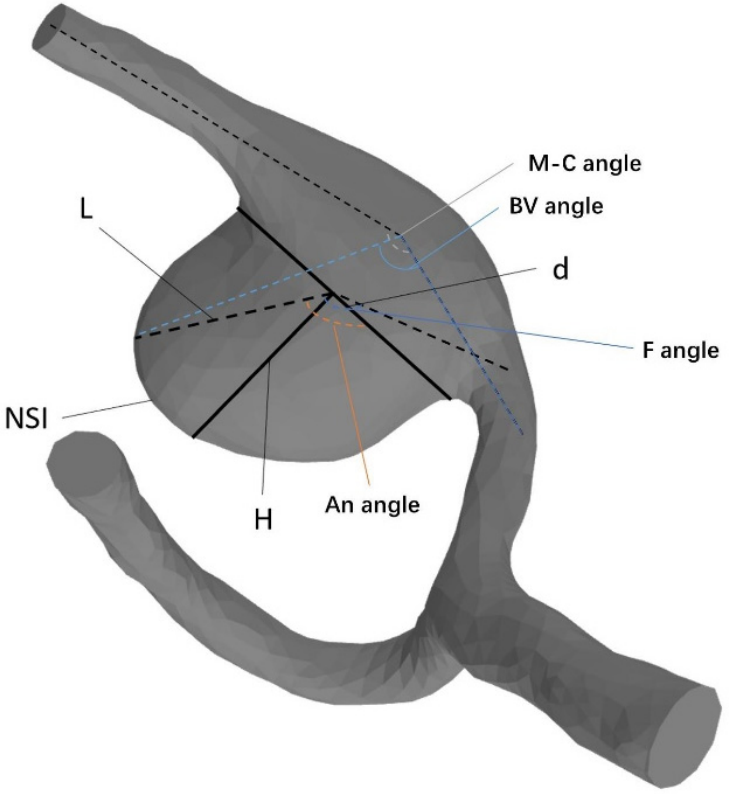

d：Aneurysm Neck Diameter

L：Maximum Height

H：Perpendicular Height

NSI：Non-Sphericity Index

F angle：Flow angle

An angle：Aneurysm angle

BV angle：Angle of blood vessel

M-C angle：Angle of blood vessel

Supplementary Figure 2: Supplementary Figure 2: Diagram illustrating aneurysm morphological parameters. (**d**) represents the average diameter at the neck. (**L**) indicates the maximum distance from the dome to the neck plane. (**H**) represents the maximum vertical distance from the dome to the neck plane. (**NSI**) Non-Sphericity Index, its calculation formula: **NSI = 1 - $(18\pi)^{1/3}V^{2/3}$/ S** (**V:** the volume of IA; **S:** the surface area of IA). (**F angle**) the angle between the vector of the blood flow of the parent artery and the aneurysm height line. (**An angle**) formed between the neck of the aneurysm and the maximum height of the aneurysm. (**BV angle**) the angle between the vector of the blood flow through the parent vessel and the vector of the blood flow through the daughter vessel. (**MC angle**) the angle between the vector of the blood flow through the parent vessel and the vector of the blood flow through the daughter vessel.

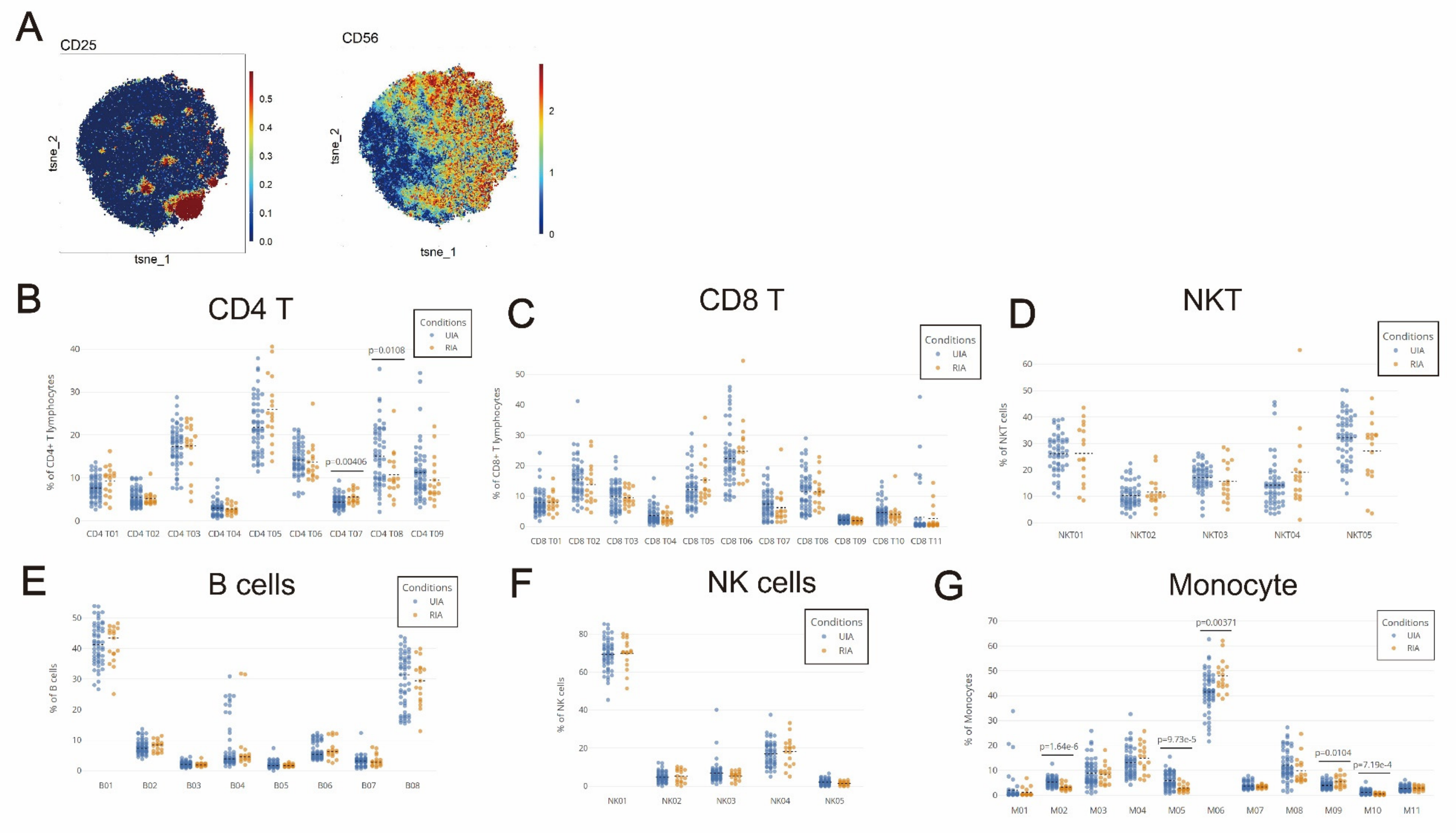

Supplementary Figure 3: CD25 and CD56 are displayed in a spectral format on the T-SNE plot (A). Scatter plots illustrate the relative proportions of each PBMC subset between the two groups (Statistically significant differences are denoted by labeled p-values) (B-G).

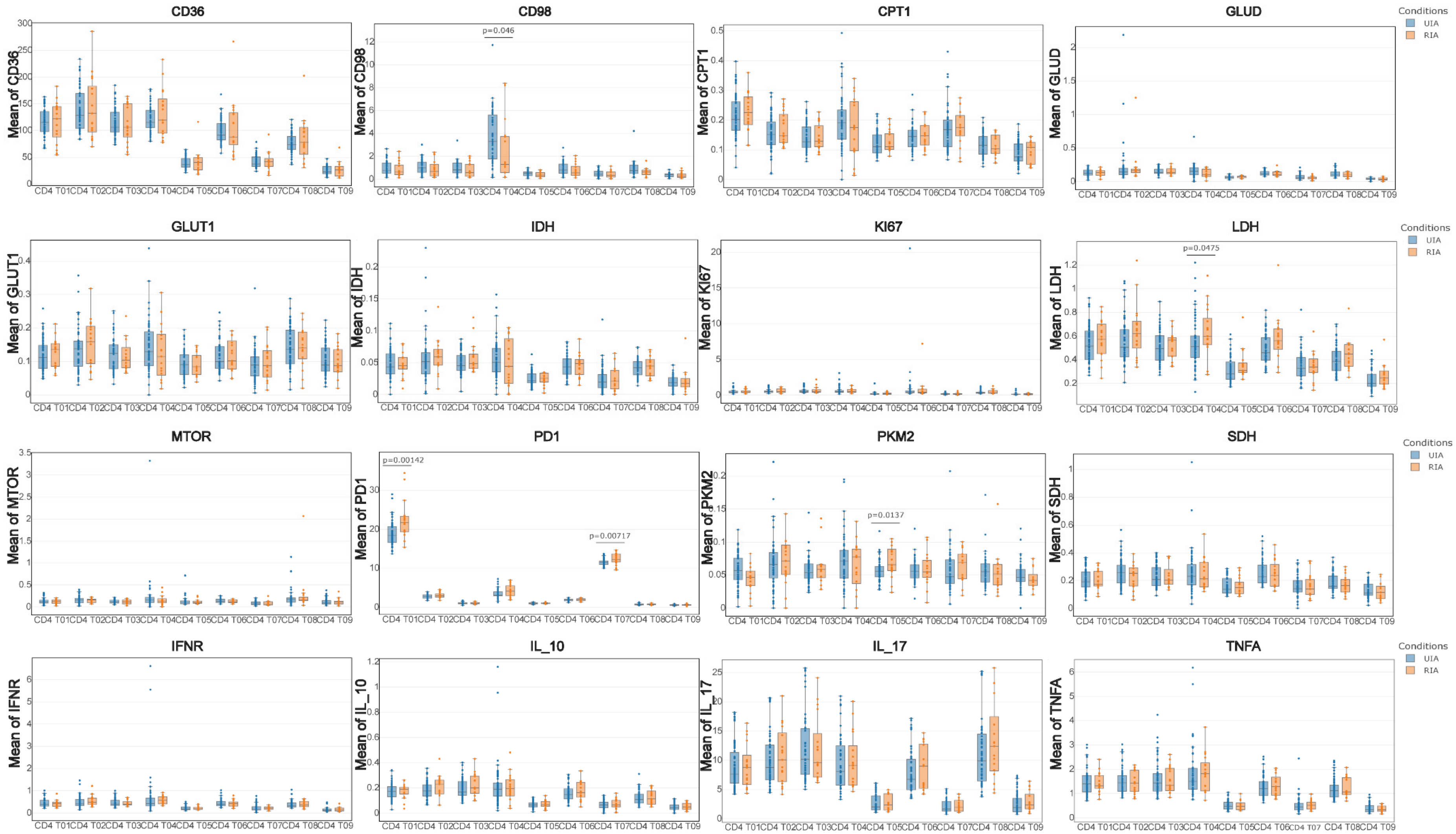

Supplementary Figure 4: Comprehensive comparison of functional and metabolic molecules among various CD4+ T cell subsets between UIA and RIA groups.

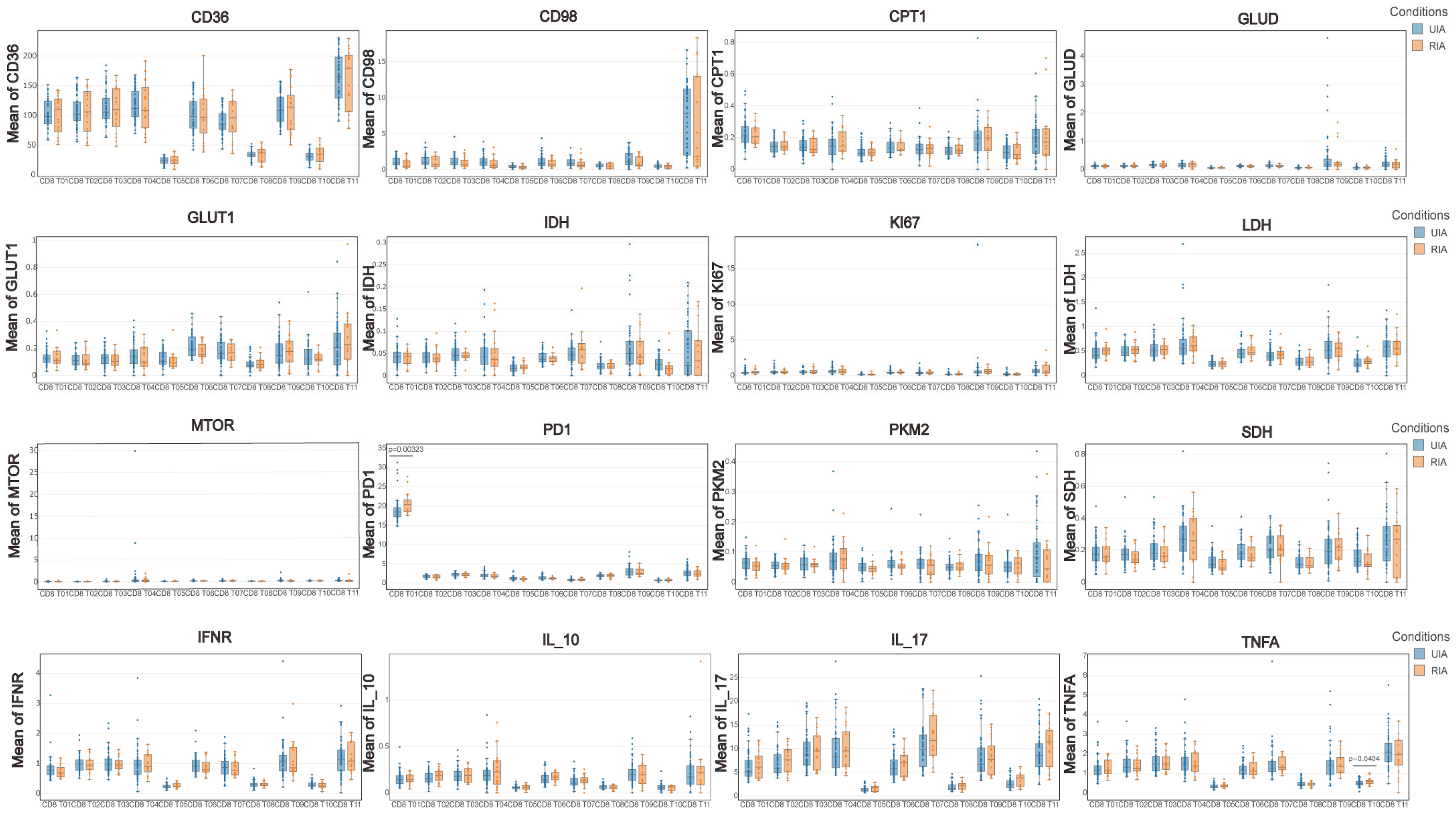

Supplementary Figure 5: Comprehensive comparison of functional and metabolic molecules among various CD8+ T cell subsets between UIA and RIA groups.

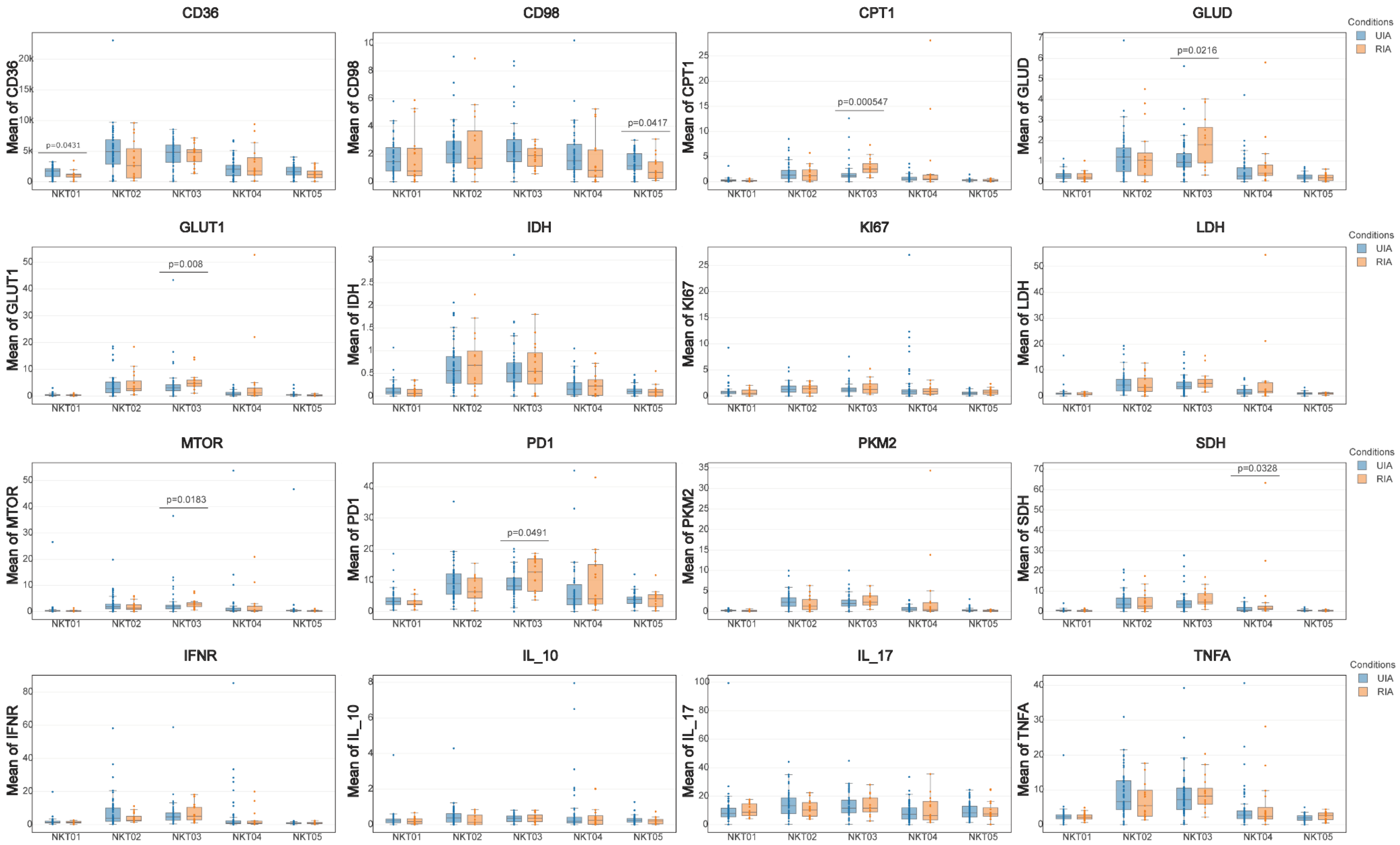

Supplementary Figure 6: Comprehensive comparison of functional and metabolic molecules among various NKT subsets between UIA and RIA groups.

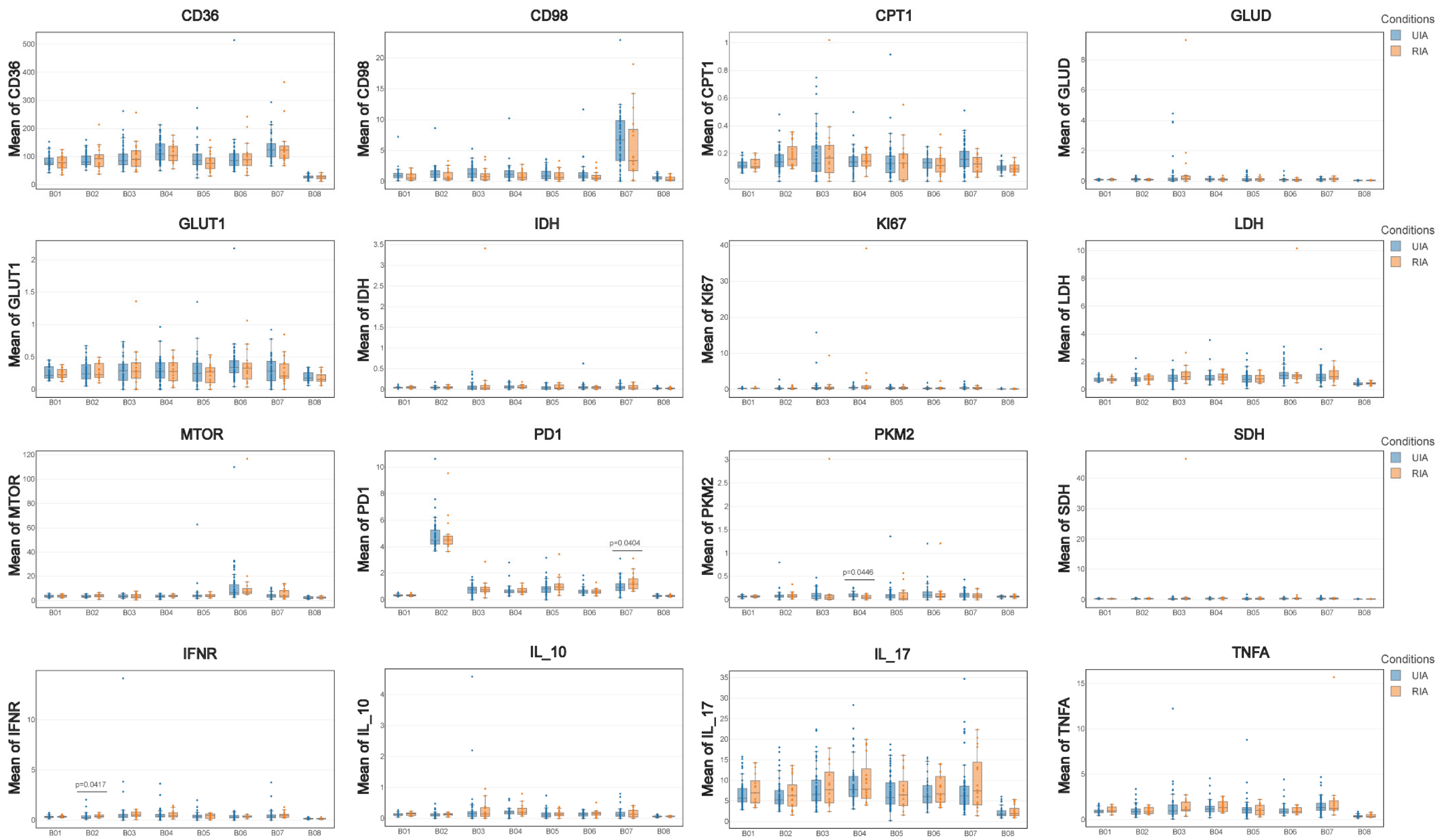

Supplementary Figure 7: Comprehensive comparison of functional and metabolic molecules among various B cell subsets between UIA and RIA groups.

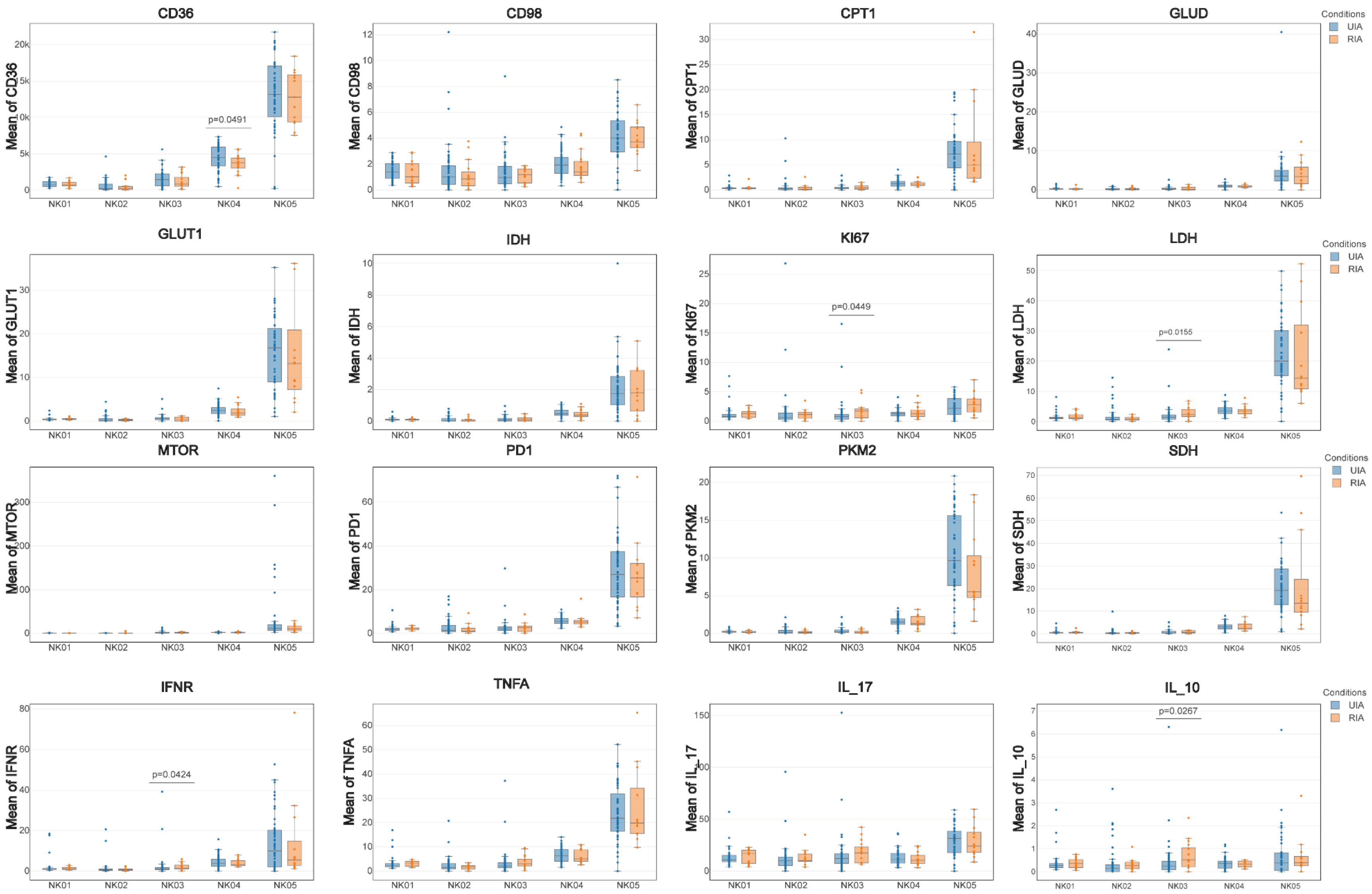

Supplementary Figure 8: Comprehensive comparison of functional and metabolic molecules among various NK subsets between UIA and RIA groups.

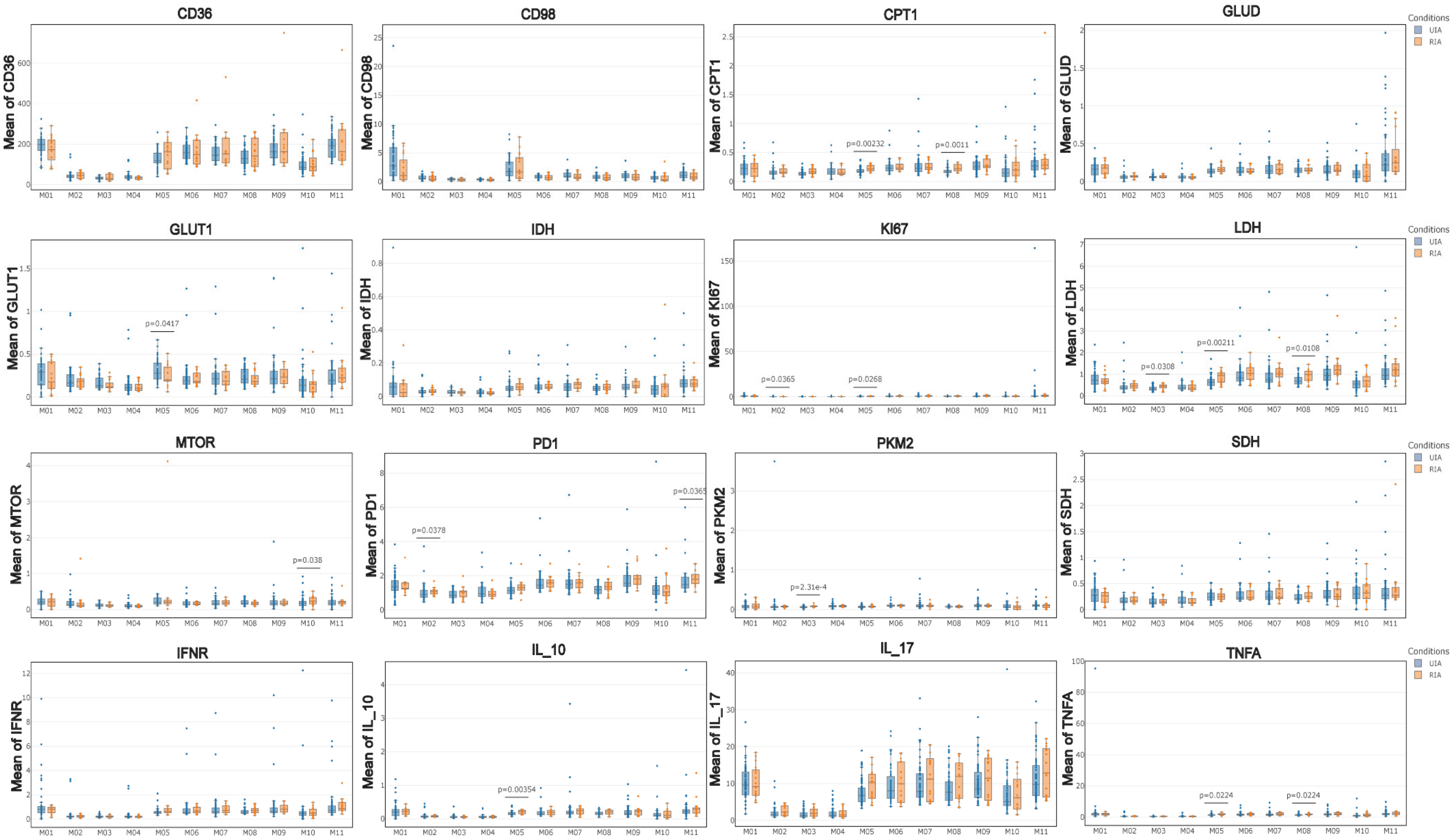

Supplementary Figure 9: Comprehensive comparison of functional and metabolic molecules among various monocyte subsets between UIA and RIA groups.

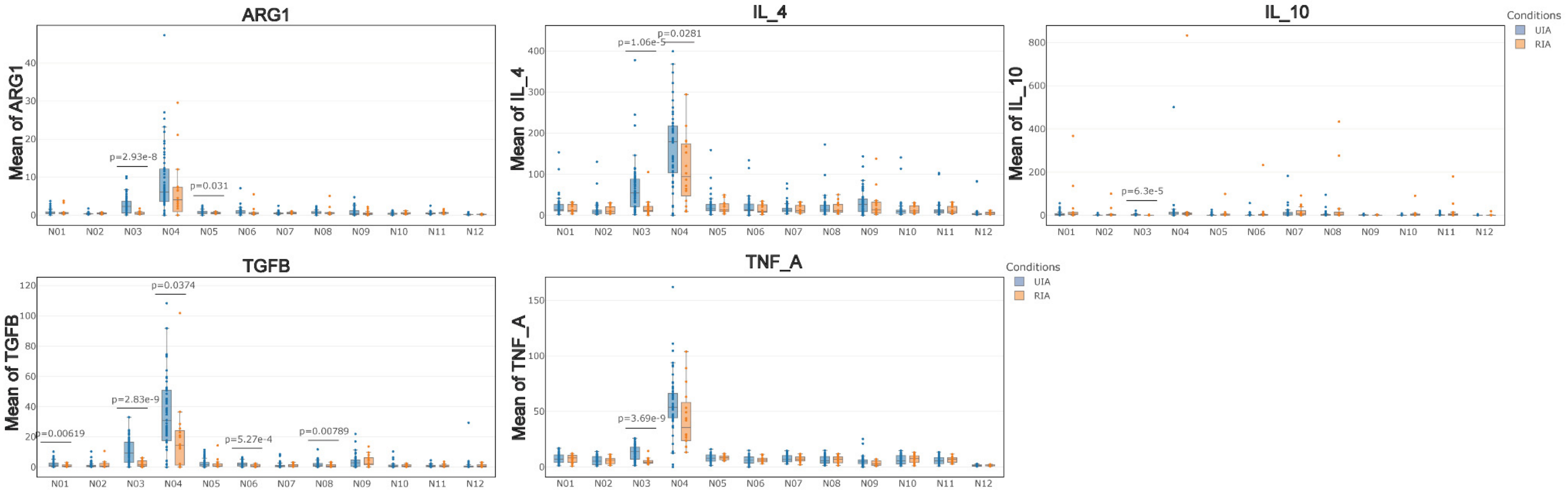

Supplementary Figure 10: Comprehensive comparison of functional molecules among various neutrophils subsets between UIA and RIA groups.

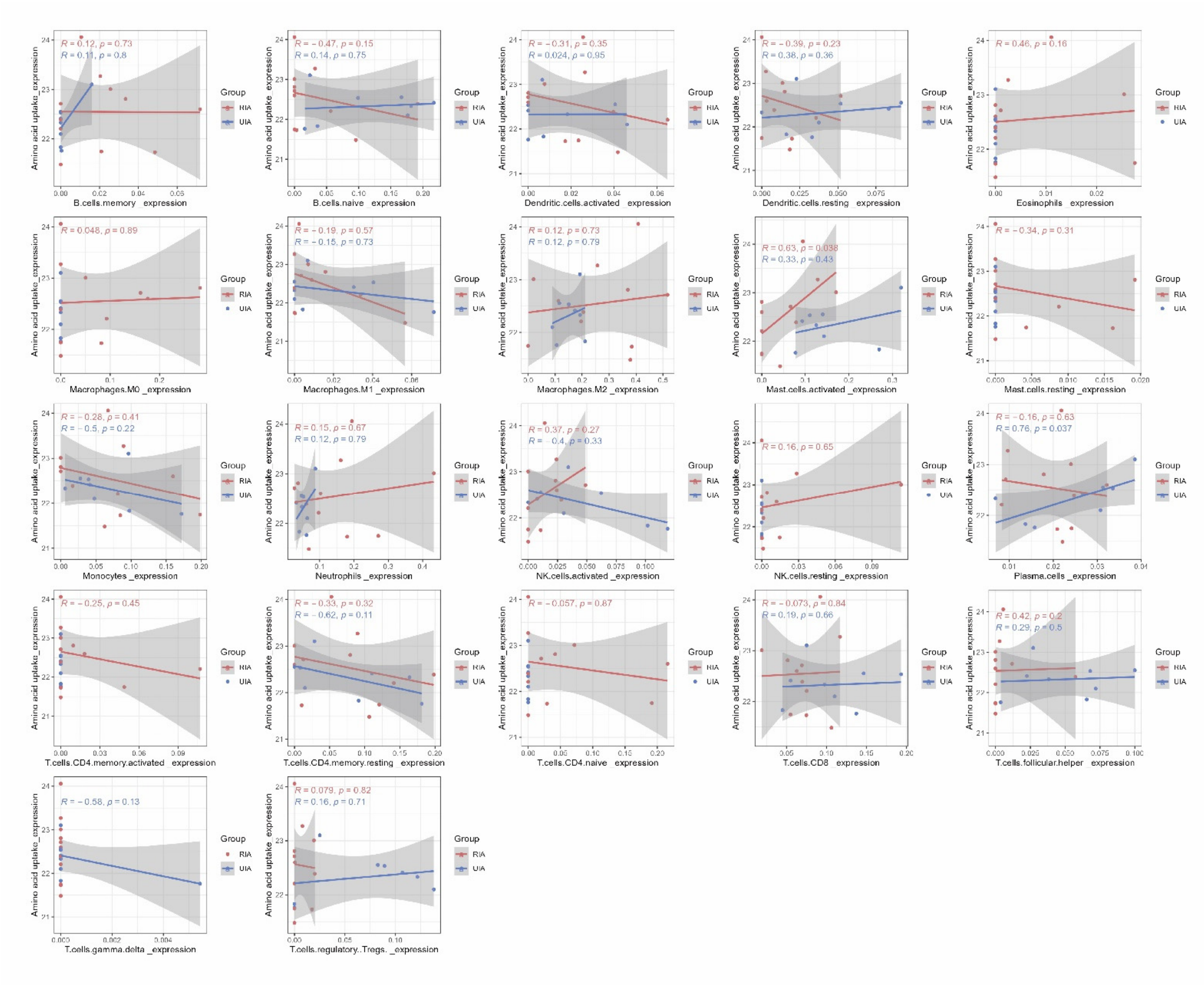

Supplementary Figure 11: Correlation analysis between 22 immune cells and protein metabolism genes.

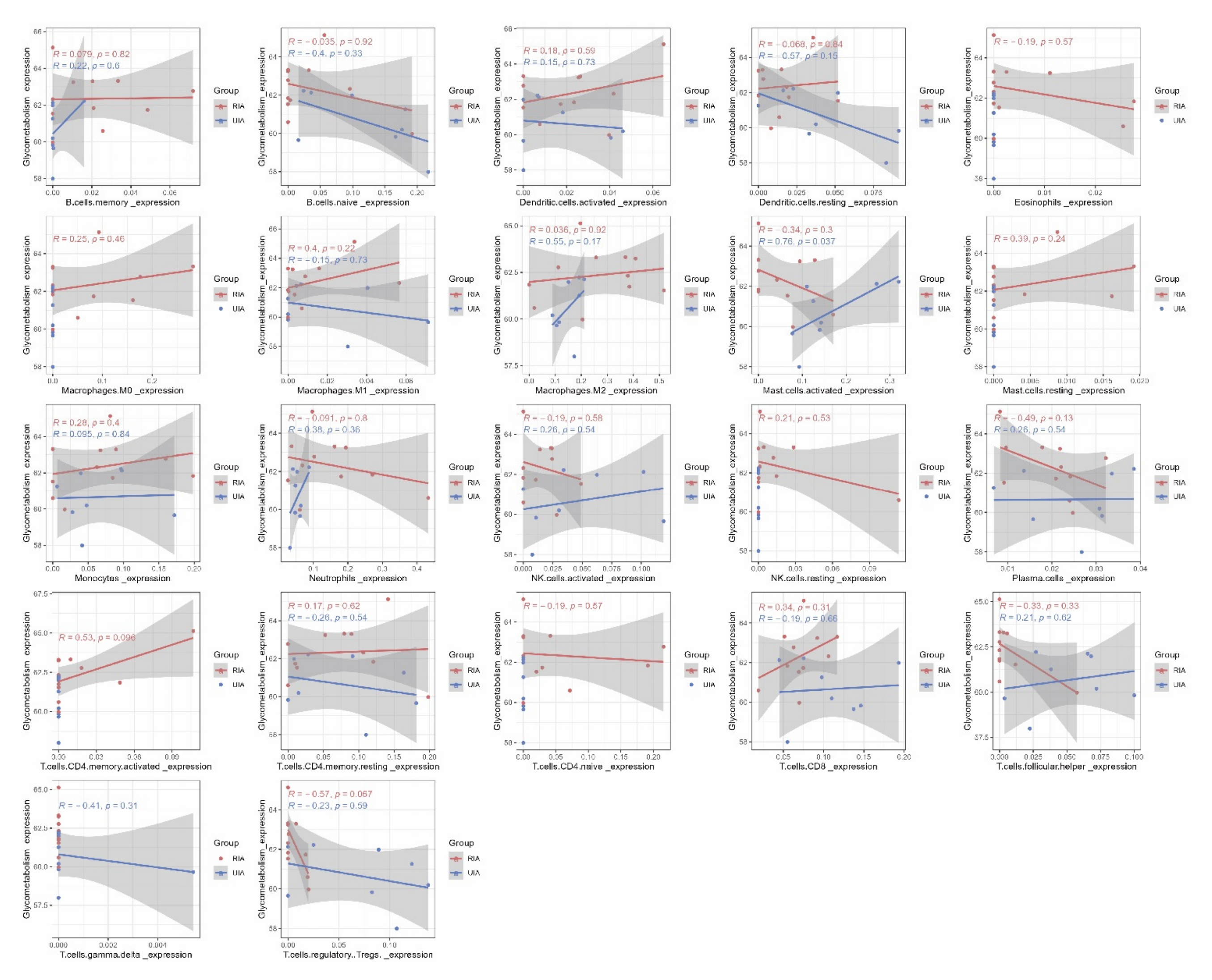

Supplementary Figure 12: Correlation analysis between 22 immune cells and glycometabolism genes.

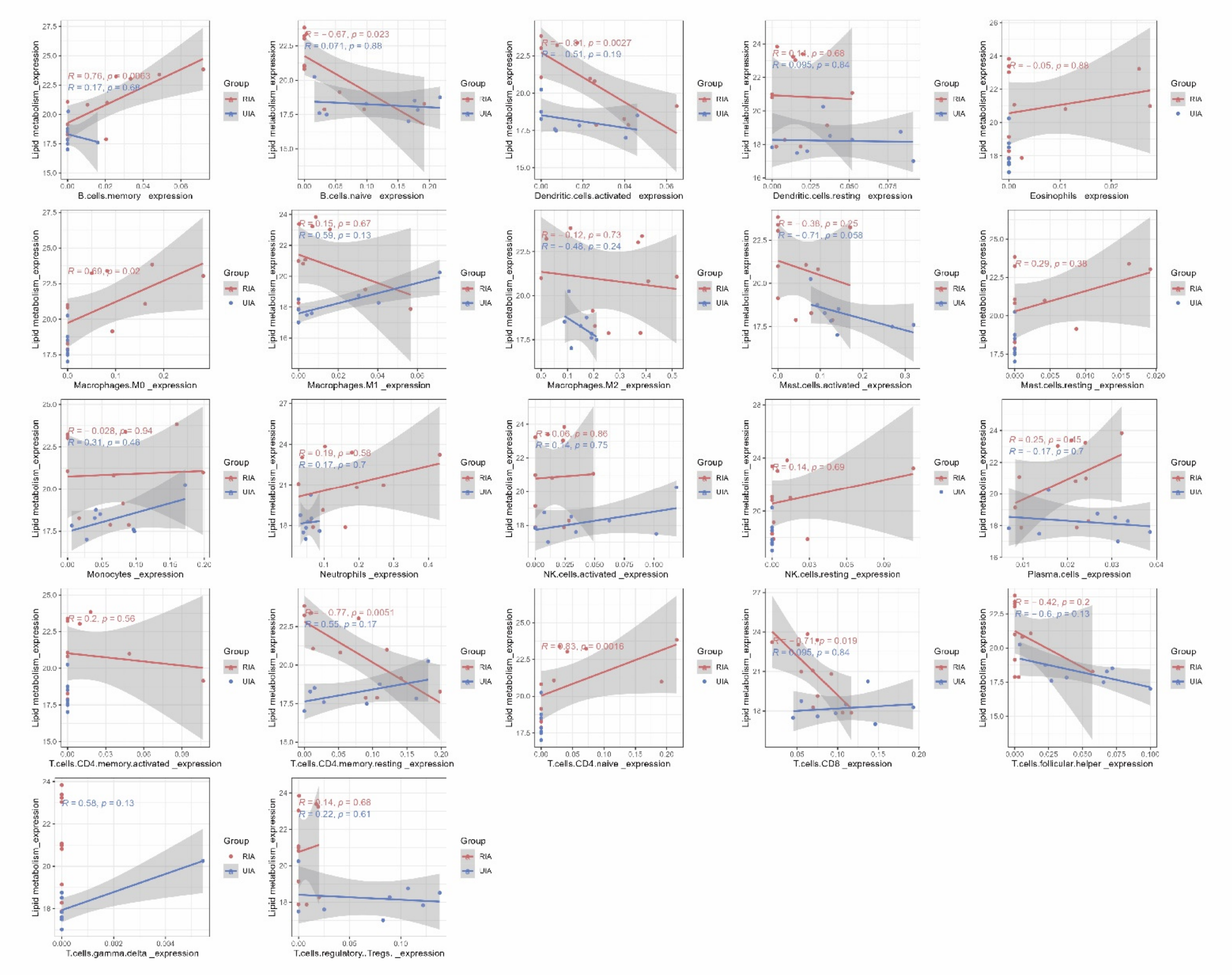

Supplementary Figure 13: Correlation analysis between 22 immune cells and lipid metabolism genes.

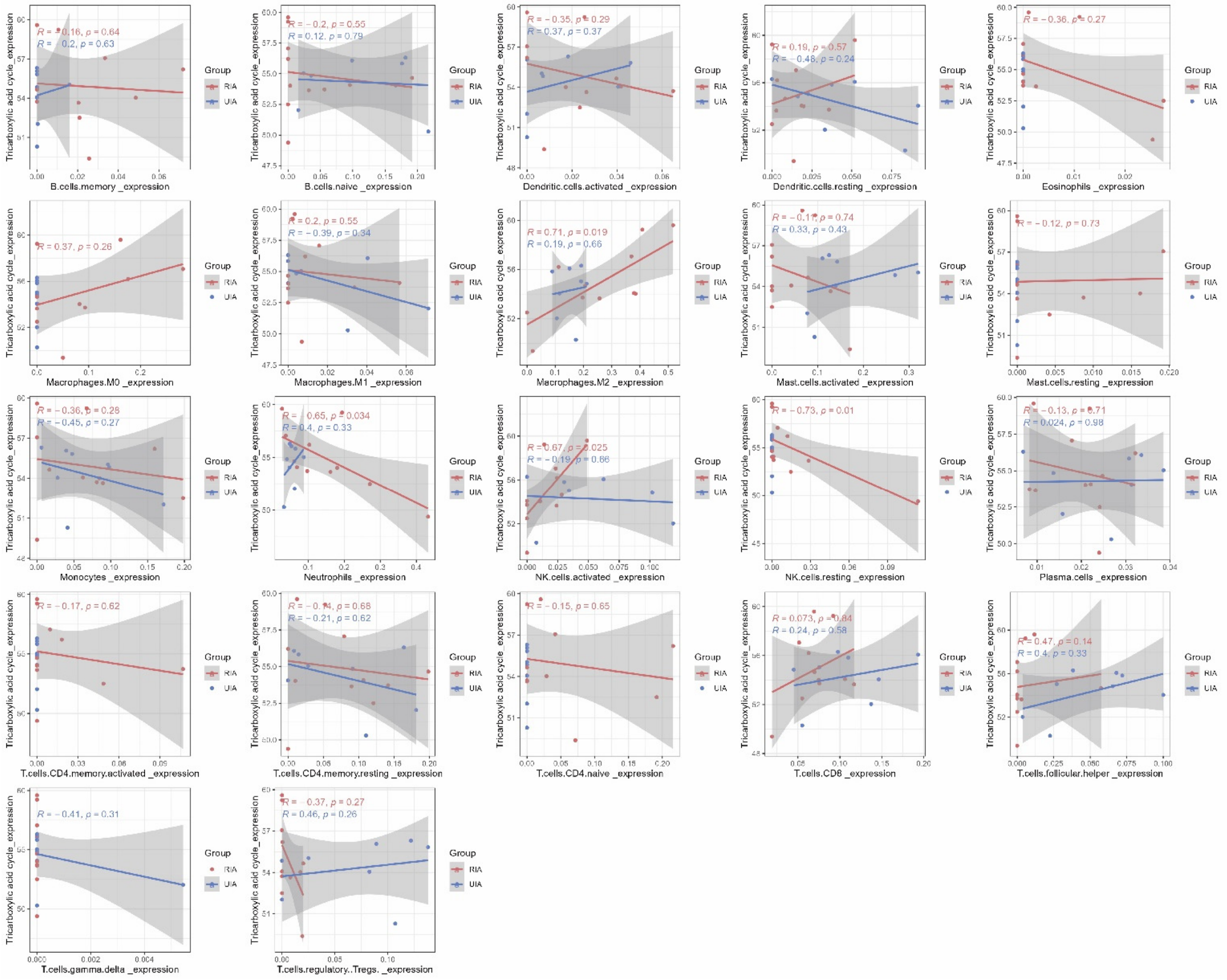

Supplementary Figure 14: Correlation analysis between 22 immune cells and tricarboxylic acid cycle genes.

Supplementary Table 1. Clinical characteristics of 72 IA patients.

Parameters	UIA (n=55)	RIA (n=17)	P-value
**Basic characteristics**			
Gender (male/female)	24/31	9/8	0.501
Age (year, Mean/IQR)	55.44/16.5	56.35/17.5	0.591
**History**			
Drinking, n(%)	10(18.18%)	4(23.53%)	0.626
Smoking, n(%)	11(20.00%)	3(17.65%)	0.830
Hypertension, n(%)	33(60.00%)	10(58.82%)	0.931
Dyslipidemia, n(%)	2(3.64%)	0(0%)	0.425
Diabetes, n(%)	8(14.55%)	3(17.65%)	0.756
**Location**			0.730
ACoA	10(18.18%)	5(29.41%)	
MCA	27(49.09%)	8(47.06%)	
PCoA	11(20.00%)	2(11.76%)	
TICA	7(12.73%)	2(11.76%)	
**Morphology**			0.711
Regular	43(78.18%)	14(82.35%)	
Irregular	12(21.82%)	3(17.65%)	

ACoA: anterior communicating artery; MCA: middle cerebral artery; PCoA: posterior communicating artery; TICA: terminal internal carotid artery

Supplementary Table 2. Clinical characteristics of 58 IA patients with imaging data.

Parameters	UIA (n=42)	RIA (n=16)	P-value
**Basic characteristics**			
Gender (male/female)	18/24	9/7	0.361
Age (year, Mean/IQR)	55.19/16.5	56.00/17.5	0.382
**History**			
Drinking, n(%)	8(19.05%)	4(25.00%)	0.617
Smoking, n(%)	9(21.43%)	3(18.75%)	0.822
Hypertension, n(%)	23(54.76%)	9(56.25%)	0.919
Dyslipidemia, n(%)	1(2.38%)	0(0%)	0.534
Diabetes, n(%)	5(11.90%)	3(18.75%)	0.499
**Location**			0.491
ACoA	6(14.29%)	5(31.25%)	
MCA	20(47.62%)	7(43.75%)	
PCoA	9(21.43%)	2(12.50%)	
TICA	7(16.67%)	2(12.50%)	
**Morphology**			0.680
Regular	32(76.19%)	13(81.25%)	
Irregular	10(23.81%)	3(18.75%)	

ACoA: anterior communicating artery; MCA: middle cerebral artery; PCoA: posterior communicating artery; TICA: terminal internal carotid artery

Supplementary Table 3. Mass cytometry antibodies panel of PBMCs.

Antigen	Symbol and Mass	Antibody clone	Source
CD45	89	HI30	Fluidigm
CD3	111	Hu113	R＆D
CD4	116	34930	R＆D
PKM2	141	EPR10138(B)	abcam
CD19	142	HIB19	Fluidigm
CCR6	143	53103	R＆D
GLUT1	145	SP168	abcam
CD45RA	146	HI100	Fluidigm
CD20	147	2H7	Fluidigm
mTOR	148	EPR427(N)	abcam
CD25	149	2A3	Fluidigm
SDH	150	EPR9043(B)	abcam
PD-1	151	913429	R＆D
CD123	152	32703	R＆D
CPT1	153	EPR21843-71-1C	abcam
LDH	154	EP1565Y	abcam
GLUD	156	EPR11369(B)	abcam
IDH	158	EPR21002	abcam
CCR7	159	G043H7	Fluidigm
TNF-a	160	EPR20972	abcam

T-bet	161	EPR27094-16	abcam
CD1c	162	L161	Biolegned
CD66b	163	913542	R&D
CD98	164	MEM-108	Biolegned
CD127	165	A019D5	Fluidigm
IL-10	166	EPR1114	abcam
GATA-3	167	EPR16651	abcam
FoxP3	169	EPR22102-37	abcam
IL-17	170	QA18A46	Biolegned
CD45RO	171	UCHL1	Biolegned
CD36	172	255606	R&D
CD56	173	301021	R&D
CD14	174	134620	R&D
CD8	175	37006	R&D
IFN-r	176	EPR23991-53	abcam
CD16	209	3G8	Fluidigm

Supplementary Table 4. Mass cytometry antibodies panel of PMNs.

Antigen	Symbol and Mass	Antibody clone	Source
CD45	89	HI30	Fluidigm
CD49d	141	9F10	Fluidigm
IL-4	142	MP4-25D2	Fluidigm
CD11b	144	ICRF44	Fluidigm
CD86	147	37301	R＆D
CD15	148	ICRF29-2	R＆D
CD34	149	581	Fluidigm
CD10	150	212504	R＆D
Arg-1	151	EPR10411	abcam
CD123	152	32703	R＆D
CD62L	153	DREG-56	Fluidigm
CD101	155	BB27	Biolegned
HLADR	156	L203	R＆D
CD33	158	WM53	Fluidigm
CD56	159	301021	R＆D
TNF-a	160	EPR20972	abcam
CD66b	163	913542	R＆D

IL10	166	EPR1114	abcam
CXCR4	167	44716	R＆D
CD14	169	134620	R＆D
CD117	170	47233	R＆D
CD38	174	S17015F	Biolegned
TGFb	176	EPR12079(B)	abcam
CD16	209	3G8	Fluidigm

Establishment and evaluation of risk prediction model for IA rupture

The China Intracranial Aneurysm Project (CIAP) is a significant ongoing initiative aimed at developing a predictive model for intracranial aneurysms. Although the CIAP focuses on many factors related to aneurysm rupture (including imaging, biomarkers, and genes), it does not include immune-related risk factors. Based on the CyTOF analysis, we found that peripheral blood of patients with RIA have the unique immune landscape. There appears to be an association between the relative proportions of various immune cell subpopulations in peripheral blood and rupture. Therefore, based on the proportions of these immune cell subgroups in peripheral blood of enrolled patients (all 72 enrolled patients), we constructed a CyTOF feature model. This model emphasizes the relationship between peripheral immune cells and the rupture, in contrast to the ongoing CIAP project. Additionally, based on the morphological parameters measured from 58 patients (42 UIA and 16 RIA) with complete preoperative imaging data, we also constructed a morphological feature model.

For the CyTOF feature model, we first used LASSO regression to select factors related to the rupture from the immune features identified by CyTOF analysis (Figure 15A-15D). The results found 8 significantly associated cell subgroups, including N01, CD4 T07, CD4 T08, M02, M05, M06, M09, and M10 (Figure 15C and 15D). Then random forest trees were used to construct the rupture prediction model. Subsequently, this model was trained by 5-fold cross-validation in the training set (30 UIA and 30RIA). The recursive feature elimination analysis showed that the model had the lowest error rate (error = 0.1284053) when four variables were included (Figure 15C). According to the weight ranking of this model variables, we selected the top four variables for optimization

(including M02, M05, M10 and M09) (Figure 15D).

The morphological model was established using a similar method as the CyTOF model. LASSO regression screened the relevant variables from the morphological parameters measured by computational fluid dynamics (CFD) (Figure 15E and 15F). The results showed that 5 morphological parameters were closely related to the rupture of intracranial aneurysms. These included d, P, BV angle, MC angle, and NSI (Figure 15G and 15H). Similarly, random forest was used to construct the prediction model, and the model was trained using a 5-fold cross-validation method in the training set (30 UIA and 30 RIA cases). The recursive feature analysis showed that the model had the lowest prediction error rate (error = 0.2181310) when two variables were included (Figure 15G). Based on the weight ranking of this model variables, the final optimization of the model was performed using the parameters d and NSI with the highest weights (Figure 15H).

The model performance was evaluated in the test set (25 UIA and 25 RIA), and the PHASES score was also included for comparison. The ROC curve showed that the CyTOF feature model (AUC=0.967) was more accurate in predicting rupture than the morphological model (AUC=0.809) and PHASES score (AUC=0.868) (Figure 15I). The OBB error plot also indicated that the prediction error of the CyTOF model was lowest (Figure 15J). In addition, calibration curves (Figure 15K) and net benefit curves (Figure 15L) were employed to assess the probability prediction quality and classification performance of these models at various probability thresholds. Overall, the results indicated that the CyTOF immune feature model demonstrated superior performance.

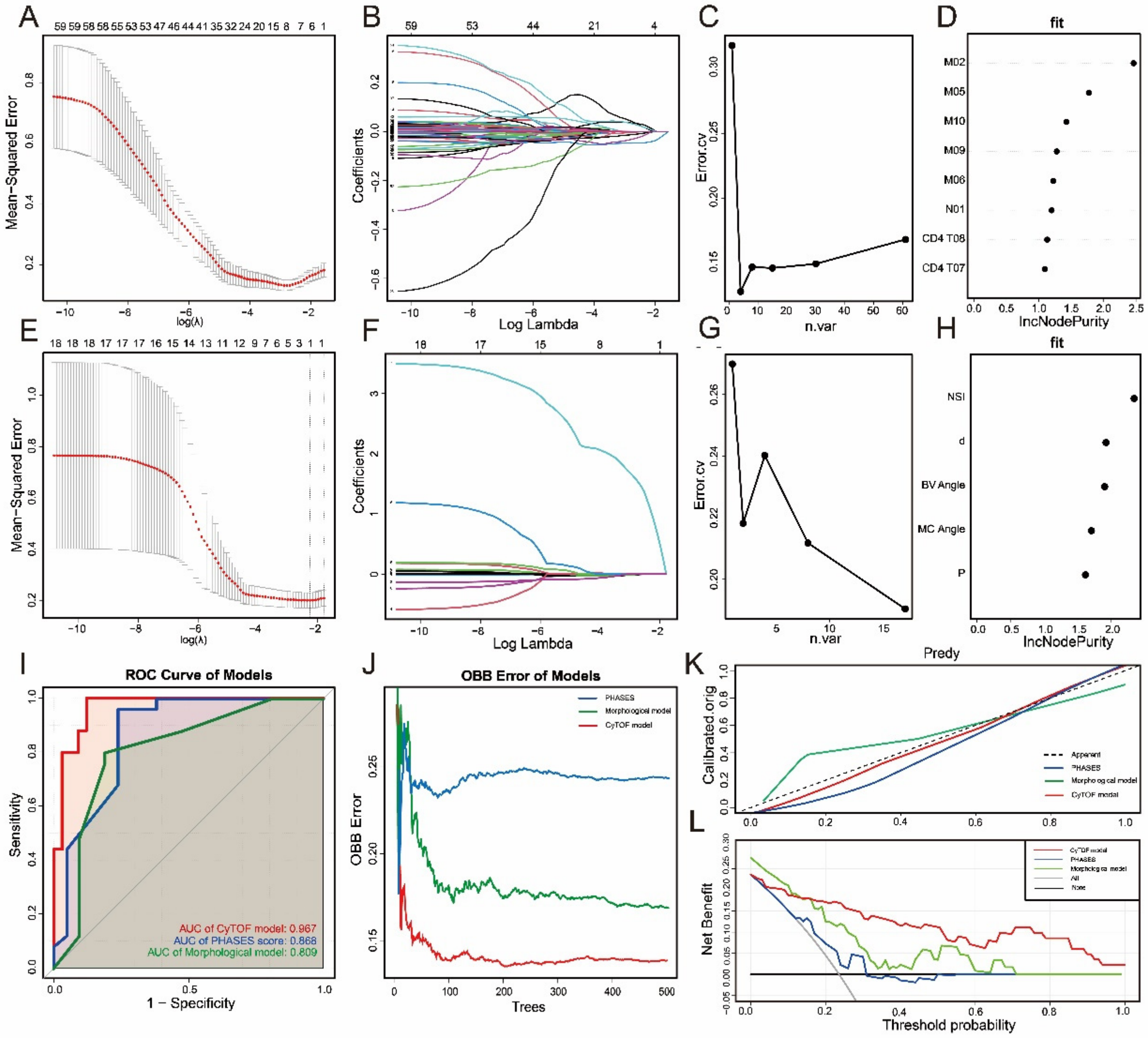

A
Mean-Squared Error
log(λ)
B
Coefficients
Log Lambda
C
Error.cv
n.var
D
fit
M02
M05
M10
M09
M06
N01
CD4 T08
CD4 T07
IncNodePurity
E
Mean-Squared Error
log(λ)
F
Coefficients
Log Lambda
G
Error.cv
n.var
H
fit
NSI
d
BV Angle
MC Angle
P
IncNodePurity
I
ROC Curve of Models
Sensitivity
1 − Specificity
AUC of CyTOF model: 0.967
AUC of PHASES score: 0.868
AUC of Morphological model: 0.809
J
OBB Error of Models
PHASES
Morphological model
CyTOF model
OBB Error
Trees
K
Predy
Calibrated.orig
Apparent
PHASES
Morphological model
CyTOF model
L
Net Benefit
Threshold probability

Supplementary Figure 15: Construction and optimization of the CyTOF feature model (A-D): The parameter plot (A) and coefficient plot (B) of LASSO regression show the variable selection process of CyTOF model. The line plot shows the optimization of the model by recursive analysis (the error is minimal when the model variables are five) (C). The scatter plot shows the weights of the selected variables by LASSO regression in model construction (D). **Construction and optimization of the aneurysm morphological model (E-H):** The parameter plot (E) and coefficient plot (F) of LASSO regression show the variable selection process of morphological model. The line plot shows the optimization of the model by recursive analysis (since LASSO regression only identified five meaningful morphological parameters, the error is minimal when the model variables are two) (G). The scatter plot shows the weights of the selected variables by LASSO regression in model construction (H). **Comparison and evaluation of the CyTOF feature model, morphological model, and PHASES score (I-L):** The ROC curve shows the predictive performance of these three optimized models in the test set (I). The OBB error plot compares the generalization abilities of the three models (J). The Calibration curve (K) and Net benefit curve (L) compare the performance of the three in different probability thresholds and the accuracy of prediction.
